# LonP1 Links Mitochondria–ER Interaction to Regulate Heart Function

**DOI:** 10.34133/research.0175

**Published:** 2023-06-16

**Authors:** Yujie Li, Dawei Huang, Lianqun Jia, Fugen Shangguan, Shiwei Gong, Linhua Lan, Zhiyin Song, Juan Xu, Chaojun Yan, Tongke Chen, Yin Tan, Yongzhang Liu, Xingxu Huang, Carolyn K. Suzuki, Zhongzhou Yang, Guanlin Yang, Bin Lu

**Affiliations:** ^1^ The Affiliated Nanhua Hospital and School of Basic Medical Sciences, Hengyang Medical School, University of South China, Hengyang, Hunan 421001, China.; ^2^ School of Laboratory Medicine and Life Sciences, Wenzhou Medical University, Wenzhou, Zhejiang 325035, China.; ^3^ National Health Commission Key Laboratory of Birth Defect Research and Prevention, Hunan Provincial Maternal and Child Health Care Hospital, Changsha, Hunan 410008, China.; ^4^ Key Laboratory of Ministry of Education for TCM Viscera-State Theory and Applications, Ministry of Education of China, Liaoning University of Traditional Chinese Medicine, Shenyang, Liaoning 110847, China.; ^5^ Key Laboratory of Diagnosis and Treatment of Severe Hepato-Pancreatic Diseases of Zhejiang Province, The First Affiliated Hospital of Wenzhou Medical University, Wenzhou, Zhejiang, China.; ^6^ Hubei Key Laboratory of Cell Homeostasis, College of Life Sciences, Wuhan University, Wuhan, Hubei 430072, China.; ^7^ Nanjing Maternity and Child Health Care Hospital, Obstetrics and Gynecology Hospital Affiliated to Nanjing Medical University, Nanjing 210004, China.; ^8^ Animal Center, Wenzhou Medical University, Wenzhou, Zhejiang 325035, China.; ^9^ Department of Cardiology, The First Affiliated Hospital of University of South China, Hengyang, Hunan, China.; ^10^ School of Life Science and Technology, Shanghai Tech University, Shanghai 201210, China.; ^11^ Department of Microbiology, Biochemistry and Molecular Genetics, New Jersey Medical School-Rutgers, The State University of New Jersey, Newark, NJ, USA.; ^12^ State Key Laboratory of Pharmaceutical Biotechnology, Department of Cardiology, Nanjing Drum Tower Hospital, The Affiliated Hospital of Nanjing University Medical School and MOE Key Laboratory of Model Animal for Disease Study, Model Animal Research Center, Nanjing University, Nanjing 210061, China.

## Abstract

Interorganelle contacts and communications are increasingly recognized to play a vital role in cellular function and homeostasis. In particular, the mitochondria–endoplasmic reticulum (ER) membrane contact site (MAM) is known to regulate ion and lipid transfer, as well as signaling and organelle dynamics. However, the regulatory mechanisms of MAM formation and their function are still elusive. Here, we identify mitochondrial Lon protease (LonP1), a highly conserved mitochondrial matrix protease, as a new MAM tethering protein. The removal of LonP1 substantially reduces MAM formation and causes mitochondrial fragmentation. Furthermore, deletion of LonP1 in the cardiomyocytes of mouse heart impairs MAM integrity and mitochondrial fusion and activates the unfolded protein response within the ER (UPR^ER^). Consequently, cardiac-specific LonP1 deficiency causes aberrant metabolic reprogramming and pathological heart remodeling. These findings demonstrate that LonP1 is a novel MAM-localized protein orchestrating MAM integrity, mitochondrial dynamics, and UPR^ER^, offering exciting new insights into the potential therapeutic strategy for heart failure.

## Introduction

Our current understanding, as well as imaging, of cellular organelles is that they are largely interdependent and form numerous membrane contact sites (MCSs) that facilitate interorganelle communication to guarantee critical cellular functions [1,2]. MCSs are hubs where 2 different organelles cooperate to execute vital functions for cellular homeostasis including ion and lipid trafficking, signaling, and organelle division.

The formation of MCSs is tightly and accurately regulated through tethering proteins positioned between 2 distinct organelles [3]. The endoplasmic reticulum (ER) is an interconnected network of tubules and flattened sacs throughout the cytoplasm. Therefore, the ER forms extensive MCSs with other organelles [2,4]. Among them, the ER–mitochondria MCS [also termed mitochondrial-associated ER membrane (MAM)] was observed and identified decades ago [5,6]. Thereafter, the structure of MAM and MAM-mediated communications between ER and mitochondria have been of profound interest for intensive studies and investigations. Currently, we know that the MAM is tethered by a plethora of molecules, such as inositol 1,4,5-triphosphate receptor (IP_3_R), voltage-dependent anion-selective channel 1 (VDAC1), mitofusin 2, and the molecular chaperone glucose-regulated protein 75 [1,7–9]. The MAM functions to transfer lipids and calcium ions between the 2 organelles, to define the sites for mitochondrial division, and to regulate cell survival [6,10]. A growing list of proteins has been identified as MAM components, but how they are recruited and function during complex cell stress situations is still poorly understood, while the participation of mitochondrial matrix proteins is largely unrecognized. Nevertheless, these factors may have important clinical implications—for example, mitochondrial changes in response to ER-stress conditions have been described in several human diseases, including type II diabetes and Alzheimer’s disease [11–13]. In addition, many studies have proved that the abnormal amount, structure, or function of MAM is related to the occurrence of cardiovascular diseases such as ischemia-reperfusion injury, diabetic cardiomyopathy, and hypertrophic cardiomyopathy [14,15]. However, the relationship between MAM and dilated cardiomyopathy (DCM) remains poorly understood.

Lon protease (LonP1) is a well-conserved mitochondrial matrix protease that selectively degrades abnormal (oxidatively modified, aggregated, etc.) proteins as a measure of protein quality control to maintain mitochondrial homeostasis [16–20]. In addition, various mitochondrial stresses including misfolded proteins and reactive oxygen species (ROS) activate the transcription of LonP1, along with mitochondrial chaperones, symbolizing an intensively characterized process called the mitochondrial unfolded protein response (UPR^mt^) [21,22]. The UPR^mt^ is a protective or adaptive transcriptional response for cell survival [23,24]. Similarly, the unfolded protein response within the ER (UPR^ER^) also causes global alteration of transcription networks to increase ER chaperones and proteases to promote the recovery of organellar protein homeostasis (proteostasis) in cells under ER stresses [25,26]. Both UPR^ER^ and UPR^mt^ are involved in the regulation of autophagy/mitophagy, metabolism, and cell survival [22,27].

Our understanding of MAM formation and their functions is far from complete. Therefore, the identification of new MAM-tethering proteins and elucidation of their regulatory mechanisms are of great importance and interest for better understanding of organelle biology and cell biology. This understanding, in turn, will substantially help interpret organ function and homeostasis, as well as the pathogenesis of chronic severe diseases, such as cardiovascular disorders and neuronal degenerative diseases.

In the present study, we first identify LonP1 as a new MAM-localized protein that links ER and mitochondria in the cardiomyocytes. We demonstrate that MAM-localized LonP1 regulates MAM formation, as the removal of LonP1 markedly reduces MAM formation and leads to mitochondrial fragmentation. Furthermore, we generate LonP1-deficient mice and reveal that the deletion of LonP1 in cardiomyocytes impairs MAM integrity and mitochondrial fusion and activates both the UPR^mt^ and UPR^ER^. Finally, our results show that LonP1 deficiency in the heart results in aberrant metabolic reprogramming and pathological heart remodeling and eventually progresses to heart failure.

## Results

### Identification of LonP1 as a new MAM-tethering protein regulating MAM integrity

LonP1 is nuclear DNA-encoded and transcribed, and the LonP1 protein is predominantly translocated to the mitochondria, where it executes surveillance monitoring of protein quality to sustain mitochondrial homeostasis. To investigate the function of LonP1 in the heart, we generated mice deficient in LonP1 specifically in the heart by mating *Lonp1^LoxP/LoxP^* mice with cardiomyocyte-specific-MHC-Cre (*αMHC-Cre*) mice (Fig. S1A). The loss of LonP1 was confirmed at both the transcript and protein levels. Levels of LonP1 mRNA and protein were extremely low and almost undetectable in the heart of LonP1 conditional knockout (cLKO) mice compared with that in littermate controls (*Lonp1^LoxP/LoxP^*; WT) after 2 weeks (Fig. S1B to D). However, we found that there was no difference in LonP1 expression in the other organs of cLKO and WT mice (Fig. S1E). Thus, our results indicate that LonP1 was efficiently and specifically deleted in the cardiomyocytes of cLKO mice.

**Fig. 1.**
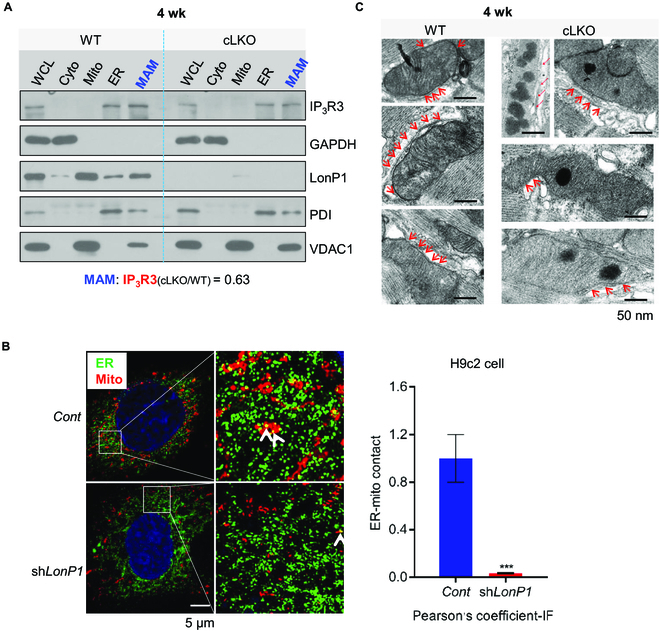
LonP1 deletion impairs MAMs. (A) Western blot analysis of the LonP1 protein level in homogenate (WCL), cytosolic (Cyto), mitochondria (Mito), ER, and MAM fractions in 4-week-old LonP1-deficient heart. VDAC1 was used as a mitochondrial marker, PDI was used as an ER marker, and IP_3_R3 was used as a marker of MAM. (B) Subcellular localization of ER and mitochondria in control and LonP1 knockdown H9c2 cells. Representative images show colocalization of mitochondria (MitoTracker Red) and the ER marker protein PDI (green). Scale bar, 5 μm. The amount of ER colocalizing with mitochondria is represented by Pearson’s correlation coefficient. Data are represented as means ± SEM (*n* = 3, ^***^P < 0.001). (C) Representative TEM images indicating the ER and mitochondrial contacts in the hearts of 4-week-old cLKO mice and their littermate controls. Arrows indicate the contacts between ER and mitochondria. Scale bars, 50 nm. See also Fig. S1. IF, immunofluorescence.

To investigate whether LonP1 is localized in other organelles, we performed conventional Percoll gradient fractionation of heart tissues to isolate organelles and MAM for Western blotting analysis. This led to the identification of LonP1 in the ER and MAM in addition to in the mitochondria (Fig. 1A). Upon deletion (cLKO), LonP1 is absent from all these organelles and MAM (Fig. 1A). Interestingly, we found that LonP1 deficiency results in a dramatic decrease (reduced to ~63% of WT) in the IP_3_R3 expression levels in the MAM fraction, suggesting the disruption of MAM integrity (Fig. 1A). Surprisingly, the mitochondrial marker protein VDAC1 was marked increased in MAM, possibly to compensate for the reduction of LonP1. Further experiments are needed to clarify this effect. To further confirm the critical role of LonP1 in the maintenance of MAM integrity of in cardiomyocytes, we knocked down LonP1 stably in H9c2 rat cardiomyocytes. Using ultrahigh-resolution microscopy, we observed a significant MAM reduction in LonP1 knockdown (sh*LonP1*) H9c2 cells compared with control H9c2 (Cont H9c2) cells (Fig. 1B). Next, we performed transmission electron microscopy (TEM) analysis of the heart tissues from control and LonP1 deletion mice. We observed many fewer contact sites in LonP1-deficient (cLKO) hearts, which indicated that the loss of LonP1 in the heart caused a significant reduction of MAM integrity (Fig. 1C). Consistently, these results reveal that LonP1 depletion in cardiomyocytes causes MAM integrity disruption and that LonP1 is indispensable in maintaining mitochondrial-ER association.

### Deletion of LonP1 leads to mitochondrial fragmentation

An important function of MAM is definition of the sites of mitochondrial fission to regulate mitochondrial dynamics in yeast and mammalian cells [10,28,29]. Mitochondrial dynamics (fission and fusion transits and balance) are critical for mitochondrial function, distribution, and turnover.

**Fig. 2.**
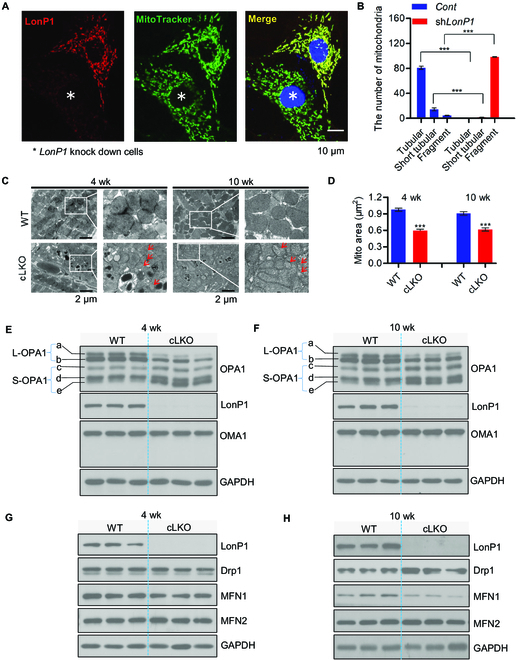
LonP1 deficiency leads to mitochondrial fragmentation. (A) The LonP1 protein levels and mitochondrial morphologies in control and LonP1 knockdown H9c2 cells were investigated by immunofluorescence with specific LonP1 antibody and MitoTracker Green. Representative confocal microscopy images are shown. Scale bar, 10 μm. Data are presented as means ± SEM (*n* = 3, ^***^P < 0.001). (B) Mitochondrial morphologies described in (A) were counted according to the criteria detailed in the Fluorescence detection in cultured cells section. (C) Representative TEM images of 4- and 10-week-old cLKO and WT hearts. Arrows indicate mitochondria with typical morphology. Scale bars, 2 μm. (D) The mitochondrial area of TEM images in (C) was quantified by ImageJ. Data are represented as means ± SEM (*n* = 3, ^***^P < 0.001). (E and F) Western blot analysis of OPA1, OMA1, and LonP1 protein levels in the heart tissue of 4-week-old (E) and 10-week-old (F) cLKO mice and WT mice. GAPDH was used as a loading control. (G and H) Western blot analysis of Drp1, MFN1, MFN2, and LonP1 protein levels in the heart tissue of 4-week-old (G) and 10-week-old (H) WT and cLKO mice. GAPDH was used as a loading control. See also Figs. S2 and S3.

LonP1 deficiency affects MAM integrity, and we investigate therefore whether this deficiency has an impact on mitochondrial dynamics. Using confocal microscopy, we observed significant mitochondrial fragmentation in sh*LonP1* H9c2 cells compared with *Cont* H9c2 cells (Fig. 2A and B and Fig. S2A). In addition, we found that mitochondria morphology and network connectivity significantly changed, including decreasing in area and perimeter, altered number of branches, and branch length in shLonP1 H9c2 cells compared with Cont H9c2 cells (Fig. S3A and B). We further confirmed that the loss of LonP1 in cardiomyocytes of 4- and 10-week-old mice caused mitochondrial fragmentation using TEM (Fig. 2C and D).

Furthermore, we observed a significant reduction in L-OPA1 (long forms of OPA1) a and b, and S-OPA1 (short forms of OPA1) c and e were increased dramatically, whereas S-OPA1 form d remained unchanged in the hearts of 4- and 10-week-old cLKO mice in comparison to control mice. OMA1 is a protease that cleaves OPA1 from L-OPA1 to S-OPA1 [30]. We observed, to our surprise, that there were no statistical significance changes in OMA1 expression in the hearts of 4- and 10-week-old cLKO mice (Fig. 2E and F). Nevertheless, cardiomyocyte-specific deletion of LonP1 increases the Yme1L expression, which also cleave OPA1 (Fig. S3C and D). Next, we examined Drp1 (dynamin related protein 1), a protein that induces mitochondrial fission, as well as MFN1 (mitofusin-1) and MFN2 (mitofusin-2), which promote mitochondrial fusion in the hearts of 4- and 10-week-old cLKO and control mice. We found that there was no difference in protein expression of Drp1, MFN1, and MFN2 in the hearts of 4-week-old cLKO and control mice, whereas the protein expression of Drp1 was statistically significantly increased, and MFN1 was statistically significantly decreased in the hearts of 10-week-old cLKO mice compared with control mice, while there were no differences in MFN2 (Fig. 2G and H). Notably, there was no difference in any of these proteins involved in mitochondrial fusion and fission in the hearts of 2-week-old cLKO mice and their littermate controls (Fig. S2B and C).

Taken together, these results indicate that LonP1 ablation in cardiomyocytes promotes OPA1 processing and Drp1 expression and reduces MFN1 expression to enhance mitochondrial fragmentation.

### Loss of LonP1 induces UPR^ER^ before UPR^mt^

**Fig. 3.**
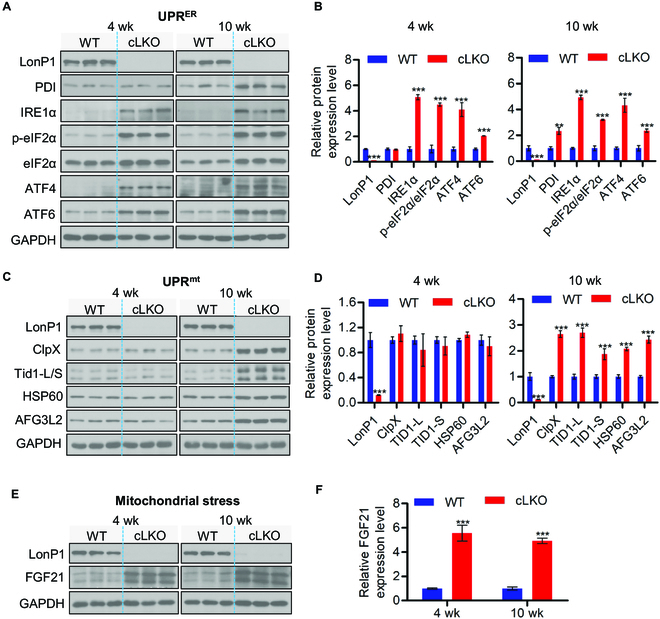
Deletion of LonP1 induces UPR^ER^ and UPR^mt^. (A and B) Western blot analysis (A) and quantification (B) of ER stress proteins PDI, IRE1α, p-eIF2α, ATF4, and ATF6 in WT and cLKO hearts at 4 and 10 weeks of age. GAPDH was used as a loading control. Data are presented as means ± SEM (*n* = 3, ^**^P < 0.01 and ^***^P < 0.001). (C and D) Western blot analysis (C) and quantification (D) of LonP1 and the UPR^mt^ related proteins ClpX, Tid1-L/S, HSP60, and AFG3L2 in WT and cLKO hearts at 4 and 10 weeks of age. GAPDH was used as a loading control. Data are represented as means ± SEM (*n* = 3, ^***^P < 0.001). (E and F) Western blot analysis and quantification of the mitochondrial stress marker FGF21 protein level in WT and cLKO hearts at 4 and 10 weeks of age. GAPDH was used as a loading control. Data are represented as means ± SEM (*n* = 3, ^***^P < 0.001). See also Fig. S4

LonP1-deficiency-mediated MAM integrity alteration may cause ER stress. To test this, we determined the expression level of UPR^ER^-related proteins in cLKO mice and controls. We observed that inositol-requiring enzyme 1α (IRE1α), eukaryotic initiation factor 2α subunit (eIF2α), phosphorylation of eIF2α (p-eIF2α), and activating transcription factors 4 and 6 (ATF4 and ATF6) increased significantly in response to LonP1 depletion in 4- and 10-week-old cLKO mice (Fig. 3A and B). In addition, we observed that protein disulfide isomerase (PDI) remained unchanged in 4-week-old cLKO and control mice, whereas the PDI protein level was significantly increased in 10-week-old cLKO mice (Fig. 3A and B).

We next examined the UPR^mt^-related proteins in cLKO and control mice. We found that ClpX, Tid1-L/S, HSP60, and the adenosine triphosphatase family gene 3-like 2 (AFG3L2) protease involved in the turnover of unfolded proteins were significantly increased in 10-week-old cLKO mice. Surprisingly, the expression levels of these UPR^mt^-related proteins remained unchanged in 4-week-old cLKO mice (Fig. 3C and D). Notably, we observed that UPR^ER^- and UPR^mt^- related protein levels remained unchanged between 2-week-old cLKO mice and their littermate controls (Fig. S4A and B). These results suggest that the cardiomyocyte-specific loss of LonP1 activates both UPR^ER^ and UPR^mt^; however, the activation of UPR^ER^ is more sensitive than that of UPR^mt^ to the loss of LonP1 in cardiomyocytes.

In addition, we evaluated the expression level of fibroblast growth factor 21 (FGF21), a marker of mitochondrial stress [31], in 4- and 10-week-old WT and cLKO mice, and we observed a strong increase in FGF21 protein levels in the hearts of 4- and 10-week-old cLKO mice (Fig. 3E and F). Similar to the case for UPR^ER^- and UPR^mt^-related proteins, there was no difference in FGF21 protein levels between 2-week-old cLKO mice and their littermate controls (Fig. S4C).

Taken together, our results reveal that LonP1 ablation in cardiomyocytes activates both UPR^ER^ and UPR^mt^; however, LonP1 deletion induces UPR^ER^ before UPR^mt^. Although LonP1 located predominantly in the mitochondrial matrix, it may play a potential pivotal role in maintaining the homeostasis of the ER and mitochondria. Our findings also indicate that cytokine FGF21 is, indeed, an early marker of mitochondrial dysfunction, suggesting that FGF21 could be a direct target of UPR^mt^.

### Cardiomyocyte-specific deletion of LonP1 leads to abnormal mitochondrial morphology and dysfunction

**Fig. 4.**
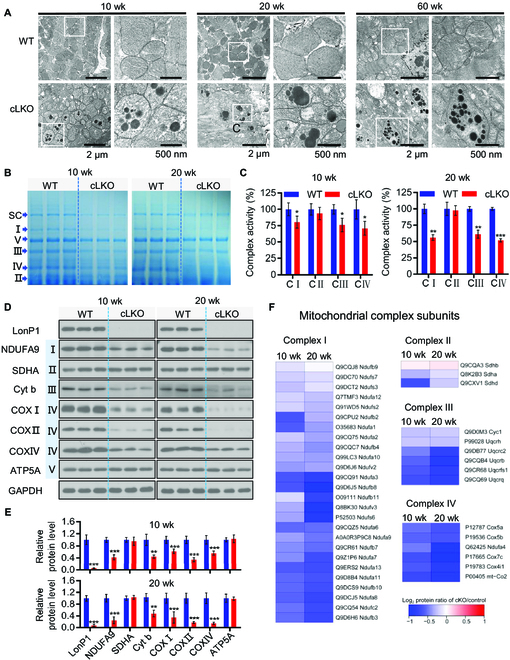
Cardiomyocyte-specific deletion of LonP1 leads to abnormal mitochondrial morphology and dysfunction. (A) TEM of 10-, 20-, and 60-week-old WT and cLKO hearts. Scale bars, 2 μm and 500 nm, respectively. (B) Coomassie Brilliant Blue staining of BN-PAGE with indicated positions and quantifications of SC (mitochondrial respiratory chain supercomplex), specific complexes I to V of WT and cLKO hearts at 10 and 20 weeks of age. (C) Enzymatic activity of complexes I to IV (C I to C IV) of the mitochondrial electron transport chain from the heart tissue of 10- and 20-week-old WT and cLKO mice. Data are represented as means ± SEM (*n* = 3, ^*^P < 0.05, ^**^P < 0.01, and ^***^P < 0.001). (D and E) Western blot analysis (D) and quantification by ImageJ (E) of protein levels for representative electron transport chain complex subunits from the heart tissue of 10- and 20-week-old WT and cLKO mice. GAPDH was used as the loading control. Data are represented as means ± SEM (*n* = 3, ^**^P < 0.01 and ^***^P < 0.001). (F) Heatmap showing protein abundance changes in the subunits of complexes I to IV obtained via high-throughput quantitative proteomics of enriched mitochondrial preparations. The relative abundance changes in WT and cLKO mice are presented using the protein ratio in relation to each control at 10 and 20 weeks of age.

We proceeded to investigate the mitochondrial morphology and respiratory chain function in the mitochondria of the hearts of cLKO mice. Similarly, TEM analysis showed increased fragment mitochondria in the LonP1 deletion hearts, which indicated abnormal mitochondrial dynamics (Fig. 4A). Moreover, we observed distorted mitochondrial morphology in the hearts of cLKO mice (Fig. 4A). We further observed extensive electron-dense aggregates within the mitochondria of cLKO mice hearts but not in other cell organelles, and no inclusions were observed in WT mice (Fig. 4A). In the heart mitochondria of 20-week-old cLKO mice, the aggregates were significantly increased. Moreover, we found that some large mitochondria were scattered, with dense deposits of different sizes filling the mitochondrial matrix, and the mitochondrial ridges significantly decreased or even disappeared in the hearts of 60-week-old cLKO mice (Fig. 4A).

We next examined the steady-state level of assembled OXPHOS (oxidative phosphorylation) complexes by blue native polyacrylamide gel electrophoresis (BN-PAGE) analysis and subsequent examination of mitochondrial respiratory chain (MRC) enzyme activities. We observed a significant decrease in the super complex and complex I, III, and IV levels in the heart tissue of both 10- and 20-week-old cLKO mice (Fig. 4B); however, the complex II and V levels only decreased slightly in the heart tissue of both 10- and 20-week-old cLKO mice (Fig. 4B). The enzymatic activities of heart tissue mitochondrial complexes I, III, and IV decreased dramatically in 10-week-old cLKO mice and further decreased in 20-week-old cLKO mice (Fig. 4C).

We also detected the protein expression level of several MRC enzyme subunits by Western blotting and carried out quantitative analysis. Consistent with the results of BN-PAGE and MRC enzyme activity analyses, we found that SDHA (succinate dehydrogenase complex flavoprotein subunit A) and alpha subunit of ATP synthase (ATP5A) encoded by the nuclear gene remain unchanged, and the mitochondrial DNA encoded subunit COX I, COX II, and Cyt b, as well as the nuclear-gene-encoded subunit NDUFA9 and COX IV, were significantly decreased, with the down-regulation in 20-week-old more apparent than that in 10-week-old mice (Fig. 4D and E). Finally, we performed quantitative proteomic analysis of the heart tissue of LonP1 knockout mice using isobaric tags for relative and absolute quantization (iTRAQ) and observed that LonP1 knockout reduced most of the mitochondrial complex I, II, III, and IV subunit expression levels (Fig. 4F). Moreover, these mitochondrial complex subunits were further reduced in 20-week-old cLKO mice, except for the 3 complex II subunits detected by iTRAQ (Fig. 4F).

These results demonstrate that LonP1 knockout in the myocardium induces the accumulation of protein aggregates, impairing mitochondrial structure and morphology, as well as MRC function.

### Loss of LonP1 in cardiomyocytes leads to metabolic reprogramming through enhancing glycogenesis and amino acid metabolism

**Fig. 5.**
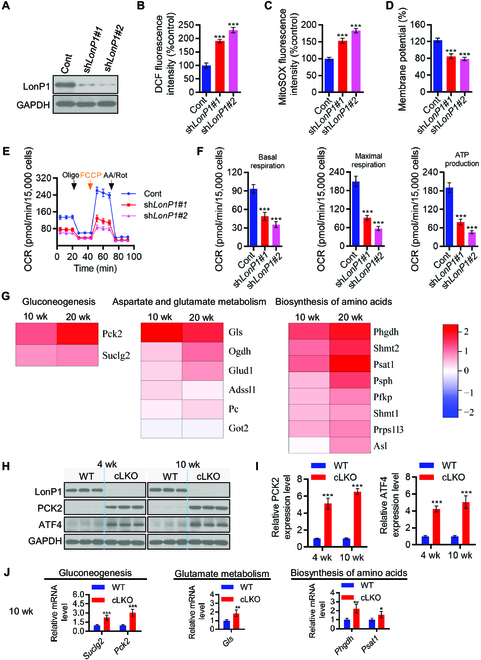
Loss of LonP1 in cardiomyocytes leads to metabolic reprogramming through enhancing glycogenesis and amino acid metabolism. (A) Western blot analysis demonstrating LonP1 stable knockdown in H9c2 cells. (B) LonP1 stable knockdown increases intracellular ROS (H_2_O_2_) production in H9c2 cells, as measured using ROS assay kit (DCFH-DA). Data are plotted as percentages of increase in the MFI and are shown as means ± SEM (*n* = 3, ^***^P < 0.001). (C) Mitochondrial superoxide levels of control and LonP1 knockdown stable H9c2 cells were detected by MitoSOX staining and analyzed by flow cytometry. Data are plotted as percentages of alteration of the MFI and are shown as means ± SEM (*n* = 3, ^***^P < 0.001). (D) LonP1 stable knockdown reduces the mitochondrial membrane potential of H9c2 cells. Cells were stained with JC-1 and analyzed by flow cytometry. The ratio of fluorescence intensities Ex/Em = 490/590 and 490/530 nm (FL590/FL530) were recorded to show the mitochondrial membrane potential level of each sample. Data are presented as means ± SEM (*n* = 3, ^***^P < 0.001). (E and F) The intact cellular OCR of H9c2 cells in the indicated conditions were measured in real time using the Seahorse XF-96 Extracellular Flux Analyzer. Basal OCRs were measured at 3 time points, followed by sequential injection of the ATP synthase inhibitor oligomycin (1 μM), the uncoupler carbonyl cyanide *p*-trifluoromethoxyphenylhydrazone (1 μM), the complex I inhibitor rotenone (1 μM), and the complex III inhibitor antimycin A (1 μM). Data are represented as means ± SEM (^***^P < 0.001). (G) Heatmap showing protein abundance changes in some proteins involved in gluconeogenesis, aspartate and glutamate metabolism, and the biosynthesis of amino acids obtained via high-throughput quantitative proteomics of enriched heart tissues. The relative abundance changes in WT and cLKO mice are expressed using the protein ratio in relation to each control at 10 and 20 weeks of age. (H and I) Western blot and quantification of representative protein levels of ATF4 and PCK2 in WT and cLKO hearts at 4 and 10 weeks, using GAPDH as the loading control. Data are represented as means ± SEM (*n* = 3, ^***^P < 0.001). (J) qRT-PCR analysis the transcripts of *Suclg2*, *Pck2*, *Gls*, *Phgdh*, and *Psat1* in WT and cLKO hearts at 10 weeks. Data are represented as means ± SEM (*n* = 3, ^*^P < 0.05, ^**^P < 0.01, and ^***^P < 0.001).

Our data showed that LonP1 depletion promoted both intracellular ROS and mitochondrial superoxide generation and caused mitochondrial depolarization (Fig. 5A to D). These results strongly indicate that LonP1 is essential for mitochondrial homeostasis and that the loss of LonP1 may cause oxidative damage in H9c2 cells. H9c2 cells are derived from fetal rat heart, which lacks a cardiomyocyte phenotype. Nevertheless, our study provides insights into the effects of deficiency of LonP1 on mitochondrial OXPHOS. Mitochondria are the major source of ATP generation, and to further evaluate the impacts of LonP1 on mitochondrial function, we analyzed cellular oxygen consumption rates (OCRs) in control and LonP1 knockdown H9c2 cells. We detected that silencing LonP1 dramatically decreased the overall OCR. We further assessed the various parameters of mitochondrial function by analyzing OCR data at each time point. Our results showed that basal respiration, maximum respiration, and ATP production were markedly decreased (Fig. 5E and F) in LonP1-knockdown H9c2 cells.

To explore the underlying molecular mechanism of the LonP1-driven H9c2 cell phenotype, we performed a quantitative proteomic analysis of heart tissue from WT and cLKO mice using iTRAQ labeling and subsequent liquid chromatography–tandem mass spectrometry analysis. iTRAQ data showed significant up-regulation of crucial metabolic enzymes, especially those participating in the processes of gluconeogenesis and aspartate and glutamate metabolism, as well as the biosynthesis of amino acids in both 10-week-old and 20-week-old cLKO mice (Fig. 5G), indicating that cardiomyocyte-specific deletion of LonP1 leads to metabolic reprogramming through enhancing glycogenesis and amino acid metabolism to promote cell survival under excessive oxidative damage stress caused by LonP1 ablation. We further confirmed that the protein levels of mitochondrial phosphoenolpyruvate (PEP) carboxykinase (PEPCK-M or PCK2) and ATF4 were increased by Western blotting analysis (Fig. 5H and I). It was well elucidated that PCK2, a key enzyme in glycogenesis, could be transcriptionally regulated by ATF4 through binding to a putative ATF/CRE composite site within the PCK2 promoter functioning as an amino acid response element, which mediates PCK2 transcriptional up-regulation [32]. Consistently, we found that the mRNA transcripts of *Suclg2*, *Pck2*, *Gls*, *Phgdh*, and *Psat1* were all up-regulated in cLKO hearts (Fig. 5J). Collectively, our data indicate that the cardiomyocyte-specific deletion of LonP1 promotes metabolic reprogramming to overcome LonP1 deficiency caused mitochondrial dysfunction, excessive oxidative stress, and intermediate metabolite limitations, thus promoting cLKO mouse survival.

### Cardiomyocyte-specific deletion of LonP1 causes pathological heart remodeling and impaired heart function

Heart failure is closely related to mitochondrial dysfunction; however, the underlying molecular mechanism remains poorly understood. Recent studies have revealed that mitochondrial ATP-dependent AAA^+^ (ATPase associated with diverse cellular activities) protease activity was significantly impaired in the mitochondria of a pressure-overload heart failure mouse model; thus, the main protease LonP1 in the mitochondrial matrix may play an important role in either resisting oxidative stress or maintaining cardiac function [33].

**Fig. 6.**
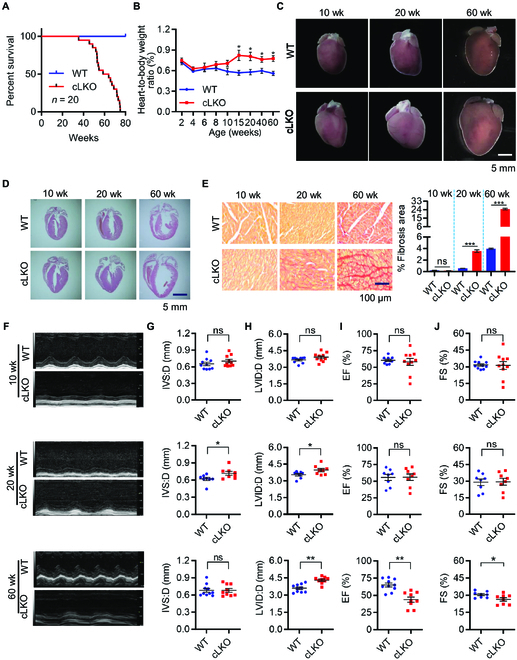
Cardiomyocyte-specific deletion of LonP1 causes pathological heart remodeling and impaired heart function. (A) Kaplan–Meier survival curves in WT and cLKO mice (*n* = 20). (B) Heart weight-to-body weight ratios in WT and cLKO mice of 2, 4, 6, 8, 10, 15, 20, 40, and 60 weeks of age (*n* = 6, ^*^P < 0.05). (C) Heart morphologies of 10-, 20-, and 60-week-old WT and cLKO mice. (D) Hematoxylin and eosin staining of WT and cLKO hearts of 10-, 20-, and 60-week-old cLKO and WT mice. (E) Representative images showing Sirius Red staining of the cardiac tissues from 10-, 20-, and 60-week-old cLKO mice and their littermate controls (left). Scale bars, 100 μm. Quantitative analysis of myocardial fibrosis in 10-, 20-, and 60-week-old WT and cLKO mice (right). Data are represented as means ± SEM (*n* = 3, ^***^P < 0.001). (F to J) Representative images of transthoracic M-mode echocardiographic tracings and echocardiographic parameters of 10-, 20-, and 60-week-old WT and cLKO mice. IVS:D, interventricular septal thickness; LVID:D, LV internal diameter in diastole; EF, ejection fraction; FS, LV fraction shortening. Data are represented as means ± SEM (*n* = 8 to 10, ^*^P < 0.05 and ^**^P < 0.01). See also Fig. S5.

To determine the effects of cardiomyocyte-specific deletion of LonP1 on the heart, we first monitored survival of in WT and cLKO mice. As shown in Fig. 6A, cLKO mice were viable but had a dramatically shortened life span (with median life span of 60 weeks). We next examined heart function from an early age up to 60 weeks of age in cLKO and WT mice. There were no significant differences observed in the heart weight-to-body weight ratio between WT and cLKO mice before 10 weeks (Fig. 6B). However, the cLKO mice at 20 weeks developed cardiomyopathy characterized by increased heart size with age (Fig. 6C and Fig. S5A and B). On the basis of hematoxylin and eosin staining of the hearts of WT and cLKO mice at different ages, we found a slight increase in the left ventricular (LV) wall thickness in 20-week-old cLKO mice and a significant increase in the cardiac size and LV dilation in 60-week-old cLKO mice (Fig. 6D and Fig. S5A and B). We observed myocardial fibrosis, as shown by Sirius Red and Masson’s trichrome staining, in the hearts of cLKO mice at 20 and 60 weeks (Fig. 6E and Fig. S5C and D).

Serial echocardiography analyses revealed progressive heart dysfunction in cLKO mice, which became apparent at 20 weeks and was characterized by a statistically significant increase in the interventricular septal depth and LV internal dimension, although the LV ejection fraction was still normal compared with the WT mice. However, cLKO mice at 60 weeks exhibited severe LV dilation, accompanied by significant ejection fraction reduction (Fig. 6F to J). In conclusion, the cardiomyocyte-specific deletion of LonP1 leads to DCM and heart failure, which reveals that LonP1 is indispensable for heart function.

## Discussion

In the present study, we identify LonP1 as a novel MAM-localized protein and uncover its critical functions in regulating mitochondrial dynamics and UPR^ER^, which are, in turn, involved in the maintenance of heart function. Cellular organelle MCSs that mediate interorganelle communications are one of the key components of organelle biology/contactology for intense investigation. These MCSs are highly dynamic and complex. However, the principles underlying MCS formation and functions are much less understood. Our identification of LonP1 as a new MCS-linking protein helps improve understanding of these principles and questions.

Our results demonstrate that LonP1-mediated ER-mitochondrial MCSs regulate mitochondrial dynamics, favoring mitochondrial fusion. The removal of LonP1 in an in vitro cell model and in vivo mouse model gave rise to consistent phenomena of mitochondrial fragmentation. Previous studies have revealed the important and interesting function of ER-mitochondrial MCSs in defining the sites for mitochondrial fission. Our findings suggest that these MCSs also promote mitochondrial fusion.

ClpP plays a critical role in the activation of the UPR^mt^ in *Caenorhabditis elegan*s [34]. It was also found that ClpX, which multimerizes with ClpP to form the functional ATP-dependent protease ClpXP, could stimulate the UPR^m^t in mammalian cells similar to the case for the UPR^mt^ in *C. elegans* [35]. A recent report showed that ClpP might be indispensable for mammalian initiation since the absence of ClpP could trigger compensatory responses in mice [36]. However, the role of ClpP in mammalian the UPR^mt^ is still unclear. LonP1 is also an ATP-dependent mitochondrial matrix protease mainly involved in the degradation of misfolded and aggregated abnormal proteins within the matrix. Here, we show that LonP1 ablation causes many compensatory responses in cLKO mice, including activation of the UPR^ER^. Strikingly, the UPR^ER^ is activated before the UPR^mt^, suggesting that LonP1 expression is a fine-tuning sensor of the UPR^ER^ and UPR^mt^. Up-regulation of both the UPR^ER^ and UPR^mt^ in cardiomyocytes of LonP1 deletion mice results in well-tolerating mice with no major changes of longevity under unstressed conditions. In addition to the activation of both the UPR^ER^ and UPR^mt^ in the hearts of cLKO mice, FGF21, a key regulator of glucose and lipid metabolism and energy balance, is also increased dramatically to compensate for the deletion of LonP1 expression. The increased expression of FGF21 further ensures cLKO mice survival under unstressed conditions.

Balanced mitochondrial dynamics are critical for normal myocardial function [37]. Mitochondrial fission ensures biogenesis and efficiently removes damaged or old mitochondria through mitophagy, whereas mitochondrial fusion facilitates the mixing and exchange of vital metabolites and mitochondrial DNA between different mitochondria to prevent the accumulation of mitochondrial damage in cells, thus properly maintaining the mitochondrial functions [38,39]. OPA1, located in the inner mitochondrial membrane of mitochondria, plays an important role in the regulation of mitochondrial fusion and fission [40]. OPA1 is cleaved by mitochondrial processing peptidase to form the mature isoform of OPA1 (L-isoform) [41], which is further processed into 2 shorter isoforms, S1 and S2 OPA1. L-OPA1 is sufficient for mitochondrial fusion; however, this feature is lost after cleavage to the S-isoform [41]. LonP1 deletion significantly reduced L-OPA1 through the cleavage of OPA1 and increased L-OPA1 levels, promoting mitochondrial fission. Moreover, Drp1, which is the master regulator of mitochondrial division in most eukaryotic organisms [40], was increased in cardiomyocyte-specific LonP1 deletion mice. These results suggest that the loss of LonP1 triggers stress-induced OPA1 processing, leads to the accumulation of S-OPA1 forms c and e, causing unbalanced fusion and fission of mitochondria, and impairs mitochondria morphology in cardiomyocytes lacking LonP1.

We demonstrate that the loss of LonP1 causes severe impairment of mitochondrial function and morphology, promoting metabolic reprogramming to meet energy limitations and intermetabolite supplies. Mitochondrial homeostasis is essential for normal cell physiological equilibrium, and its disruption promotes mitophagy, which contributes to multiple diseases [42]. Our results clearly show that LonP1 ablation in myocardial cells promotes gluconeogenesis and aspartate and glutamate metabolism, as well as the biosynthesis of amino acids, via up-regulating multiple vital metabolic catalytic enzymes, such as SUCLG2, GLS, PHGDH, and PAST1. Importantly, PCK2, a key enzyme of gluconeogenesis, is dramatically transcriptionally up-regulated because of LonP1 depletion, suggesting that LonP1 ablation may drive PCK2-mediated metabolic adaptation in cardiac myocytes to support tricarboxylic acid (TCA) cycle metabolism and glycolytic intermediates for biosynthesis [43]. The activity of PCK2 depends on mitochondrial guanosine 5′-triphosphate generated by the SUCLG2 form of SCS (succinyl-CoA synthase) in the mitochondrial matrix [44]. We show that the SUCLG2 protein and mRNA expressions are stimulated by LonP1 ablation, indicating a synergistic effect to strengthen PCK2 activity in LonP1-deficient cardiomyocytes. Moreover, we found that ATF4 was up-regulated, indicating that PCK2 could be transcriptionally regulated by ATF4 through regulating a putative ATF/CRE composite site containing the PCK2 promoter, which caused transcriptional up-regulation of PCK2 [32,45]. Our results argue that ATF4-induced PCK2 expression could promote LonP1 deletion cardiomyocytes survival in energy- and intermediate metabolite-limited environments. These results suggest that LonP1 deletion cardiomyocytes may activate ATF4/PCK2-mediated metabolic reprogramming to orchestrate energy and intermediate metabolite homeostasis, which contributes to myocardial cell survival.

In the past, many puzzling results have been reported concerning ER stress induced by mitochondrial dysfunction/stress. Frequently, researchers reveal the mitochondrial structural or functional defects induced by ER stress without a reasonable explanation. Here, we unveiled that LonP1-mediated MAMs control the UPR^ER^, and this finding may provide a clue to this long-standing obscurity (Fig. S6). As the reduction of LonP1 protein in cardiac myocytes leads to DCM and heart failure, it is possible that increased expression of LonP1 may have a cardioprotective effect against DCM, preventing heart failure. It will also be interesting to examine whether there is a correlation between the cardiac level of LonP1 and the incidence of DCM in humans.

## Materials and Methods

### Mice

All mice used in this study were housed at the Wenzhou Medical University Animal Center in a specific pathogen-free facility with individually ventilated cages, and both sexes are used. The room in the animal facility has controlled constant temperature (20 to 25 °C) and humidity (30% to 70%), with a 12-h light/dark cycle. Mice were provided ad libitum access to water and normal rodent chow diet. All procedures were reviewed and approved by the Institutional Animal Research Committee of Wenzhou Medical University.

### Reagents and antibodies

Glucose was obtained from Sigma-Aldrich (G7021). The intact cellular OCR assay kit was purchased from Agilent Technologies (103015-100). Horseradish-peroxidase-conjugated, anti-rabbit (A0208), and anti-mouse (A0216) immunoglobulin G, the ROS assay kit [2′,7′-dichlorodihydrofluorescein diacetate (DCFH-DA),S0033S], and 5,5′,6,6′-tetrachloro-1,1′,3,3′-tetraethylbenzimidazolylcarbocyanine iodide (JC-1, C2005) were obtained from Beyotime. The BCA Protein Assay Kit (23227) and Pierce ECL Western Blotting Substrate (32106) were obtained from Thermo Fisher Scientific. Primary antibodies were obtained from the following suppliers: anti-NDUFA9 (20312-1-AP, Proteintech), anti-Cyt b (55090-1-AP, Proteintech), anti-Cox II (55070-1-AP, Proteintech), anti-ATP5A (14676-1-AP, Proteintech), anti-MFN1 (13798-1-AP, Proteintech), anti-MFN2 (12186-1-AP, Proteintech), anti-AFG3L2 (14631-1-AP, Proteintech), anti-glyceraldehyde-3-phosphate dehydrogenase (GAPDH; M20028, Abmart), anti-LonP1 (28020S, Cell Signaling Technology), anti-HSP60 (12165, Cell Signaling Technology), anti-PDI (3501P, Cell Signaling Technology), anti-IRE1α (3294P, Cell Signaling Technology), anti-eIF2α (5324S, Cell Signaling Technology), anti-p-eIF2α (3597S, Cell Signaling Technology), anti-ATF4 (11815s, Cell Signaling Technology), anti-ATF6 (65880, Cell Signaling Technology), anti-PCK2 (6924, Cell Signaling Technology), anti-VDAC1 (4661S, Cell Signaling Technology), anti-IP_3_R3 (610313, BD Pharmingen), anti-Drp1 (611113, BD Pharmingen), anti-OPA1 (612607, BD Pharmingen), anti-Tid1-L/S (sc-18820, Santa Cruz Biotechnology), anti-OMA1 (sc-515788, Santa Cruz Biotechnology), anti-SDHA (ab14715, Abcam), anti-Cox I (ab14705, Abcam), anti-Cox IV (ab1460643, Abcam), anti-ClpX (ab168338, Abcam), and anti-FGF21 (ab171941, Abcam). Protease (Complete Mini, catalog no. 11 836 145 001) and phosphatase (PhosphoSTOP, catalog no. 04 906 837 001) inhibitor cocktail tablets were purchased from Roche Applied Science. MitoTracker Green FM (9074) was purchased from Cell Signaling Technology. 4′,6-Diamidino-2-phenylindole (DAPI; catalog no. P-36935) and MitoTracker Red CMXRos (M7512) were obtained from Thermo Fisher Scientific. The Sirius Red staining kit (ab150681) was purchased from Abcam.

### Cell lines and cell culture

The human low-passage human embryonic kidney (HEK) 293T cell line was obtained from the Cell Bank of the Chinese Academy of Sciences. The H9c2 rat embryonic cardiomyocyte cell line was obtained from the Model Animal Research Center of Nanjing University. HEK293T and H9c2 cells were cultured in Dulbecco’s modified Eagle’s medium (DMEM; #11960044, Life Technologies) supplemented with 10% fetal bovine serum (FBS; #12484028, Life Technologies) and antibiotics [penicillin (100 U/ml) and streptomycin (100 μg/ml)] at 37 °C in a humidified incubator with 5% CO_2_. LonP1 stable knockdown and control H9c2 cells were cultured in DMEM supplemented with 10% FBS, penicillin, streptomycin, and puromycin (2.0 μg/ml) in a humidified incubator with 5% CO_2_. Both cell lines were confirmed as mycoplasma-free and authenticated by the Cell Bank of the Chinese Academy of Sciences and the Model Animal Research Center of Nanjing University before sending to our laboratory. Cell lines were also routinely tested and confirmed to be mycoplasma-free during this study.

### Hematoxylin and eosin staining

Heart tissues were fixed, embedded in paraffin, and sectioned following standard protocols. The sections were stained with hematoxylin for 15 min and washed in running tap water for 20 min. The sections were then counterstained with eosin from 30 s to 1 min. Finally, the sections were dehydrated in 95% and absolute ethanol and mounted in neutral balsam (#BL704A, Biosharp).

### Sirius Red staining

Sirius Red staining was performed using a Picro Sirius Red staining kit according to the manufacturer’s instructions (ab150681, Abcam). Briefly, paraffin-embedded heart sections were deparaffinized/hydrated, and a series of washes was performed, followed by staining with Picro’s Sirius Red for 1 h and washing for 1 min in 0.1 N of HCl. Slides were dehydrated and mounted in synthetic resin.

### Echocardiography

An experienced researcher blinded to the study performed echocardiographic evaluations to avoid biases. A Vevo 3100 high-resolution microultrasound system (FUJIFILM Visual Sonics Inc.) was used to determine heart function and ventricular dimensions. For this procedure, 1.5% isoflurane was used to anesthetize mice, and then the mice were placed on a heating table in a supine position. M-mode and 2-dimensional (2D) images were recorded along a short-axis view from the mid-left ventricle at the tips of the papillary muscles. The interventricular septal thickness and LV internal diameter were measured at end-diastole and end-systole. The fractional shortening and ejection fraction were calculated from the LV dimensions in the 2D short-axis view.

### Transmission electron microscopy

The heart tissue samples were fixed with 2.5% (v/v) glutaraldehyde buffer for 15 min at room temperature, followed by overnight at 4 °C. The following day, the samples were treated with 1% osmium tetroxide and 0.1 M cacodylate buffer for 1 h. Samples were stained in 1% uranyl acetate and dehydrated with ethanol. Epoxy-resin-embedded samples were sectioned and placed on formvar/carbon-coated copper grids. Grids were stained with uranyl acetate and lead nitrate. Then, the samples were examined with a JEM1400 TEM (JEOL).

### Isolation of mitochondria-associated membranes

The isolation of MAM was performed following a modified published protocol [46]. Briefly, heart tissues were homogenized in a Dounce homogenizer at 4 °C. Several steps of centrifugation at 4 °C were performed to obtain cytosolic, ER, and crude mitochondrial fractions. To obtain purified MAM, the mitochondrial suspension was fractionated in Percoll medium at 95,000*g* for 30 min at 4 °C (SW40 rotor, Beckman, Fullerton, CA, USA). The MAM fraction was collected from the Percoll gradient with a Pasteur pipette and diluted 10 times with prechilled mitochondria resuspending buffer. The MAM suspension was subsequently centrifuged 3 times at 6,300*g* for 10 min at 4 °C, and the supernatant was collected each time. Next, the MAM supernatant was transferred to new tubes and centrifuged at 100,000*g* for 1 h at 4 °C (70-Ti rotor, Beckman). Finally, the obtained subcellular fraction was analyzed by Western blotting.

### Isolation and purification of mitochondria

Heart tissues were separated and washed in buffer A [0.22 M mannitol, 0.075 M sucrose, and 30 mM tris-HCl (pH 7.4)] and then homogenized in buffer B [0.22 M mannitol, 0.075 M sucrose, 30 mM tris-HCl, 75 mM bovine serum albumin, and 0.5 mM EGTA (pH 7.4)] by 50 to 100 strokes in a tight-fitting Dounce homogenizer [47]. The lysate was centrifuged at 750*g* for 10 min at 4 °C. Next, the supernatant was centrifuged at 9,000*g* for 15 min at 4 °C, and the precipitate was washed twice with buffer C [0.22 M mannitol, 0.075 M sucrose, 30 mM tris-HCl, and 75 mM bovine serum albumin (pH 7.4)] and then centrifuged at 10,000*g* for 15 min. The precipitate in the tube was the crude mitochondria, and this mitochondrial fraction was suspended in 200 μl of buffer D [10 mM tris-HCl (pH 7.4), 1 mM EDTA, and 0.32 M sucrose] and 2 μl 100 mM phenylmethylsulfonyl fluoride.

### BN-PAGE gel

BN-PAGE gel was performed using a previously published protocol with modifications [48]. Briefly, buffer [50 mM NaCl, 50 mM imidazole, 2 mM 6-aminohexanoic, and 1 mM EDTA (pH 7.0)] was added to 400 μg of pelleted mitochondria. Then, 12 μl of digitonin was added 20% (w/v), and the mitochondria were solubilized for 10 to 20 min. After centrifugation at 20,000*g* for 45 min, the supernatants were collected. Next, we added a mixture of glycerol/Coomassie blue G-250 dye (2:1) to the samples to yield a sample/mixture ratio of 5:1 (v/v). Finally, the samples were run on 3% to 11% acrylamide gradient gels at 4 °C.

### Western blot analysis

Tissue samples were washed 3 times with ice-cold phosphate-buffered saline (PBS) and homogenized using a homogenizer (Kinematica AG) in 1.5 ml of tissue radioimmunoprecipitation assay lysis buffer [50 mM tris-HCl (pH 7.4), 1.0% Triton X-100, 1% sodium deoxycholate, 0.1% SDS, and 150 mM NaCl] supplemented with a protease inhibitor cocktail tablet, and PhosSTOP phosphatase inhibitor cocktail tablet. Tissue homogenates were cleared by centrifugation at 18,000*g* for 25 min at 4 °C, and the supernatants were collected in clean microcentrifuge tubes on ice. A similar procedure was used to prepare whole-cell extracts from cells. Briefly, cells were washed with ice-cold PBS and lysed in radioimmunoprecipitation assay lysis buffer supplemented with protease and phosphatase inhibitors on ice for 20 min, followed by centrifugation at 18,000*g* for 30 min at 4 °C, and the supernatants were collected. Protein concentrations of the tissue homogenates or whole-cell extracts were determined using the Pierce BCA protein assay kit.

Tissue or cell extracts equivalent to 20 μg of total protein were resolved in 10% SDS-PAGE gels, followed by electrophoretic transfer onto a nitrocellulose membrane (#1620097, Bio-Rad) in tris-glycine buffer. Blots were blocked at room temperature for 1.5 h in 5% nonfat milk in tris-buffered saline-Tween 20 (TBST) on a shaker and then incubated with the primary antibodies as indicated in 5% nonfat milk TBST overnight at 4 °C. The membrane was washed in TBST at least 3 times for 10 min and then incubated with horseradish-peroxidase-conjugated anti-rabbit or anti-mouse immunoglobulin G at room temperature for 1 h with gentle shaking. Immunoreactive proteins were detected by enhanced chemiluminesence reagent according to the manufacturer’s protocol. The optical density of the Western blot signals was quantified using the National Institutes of Health (USA) ImageJ software.

### RNA preparation and quantitative real-time polymerase chain reaction

The total RNA was extracted from heart tissues using TRIzol (15596026, Thermo Fisher Scientific) according to the manufacturer’s instructions. The total RNA (1 μg) from each ample was used to synthesize first-strand cDNA by reverse transcription using the PrimeScript RT reagent Kit with gDNA Eraser (RR047A, Takara), according to the manufacturer’s instructions. The cDNA produced was subsequently used as a template for quantitative real-time polymerase chain reaction (qRT-PCR). qRT-PCR analysis, which was performed using a 2-μl cDNA/20-μl reaction volume on the CFX Connect Real-Time PCR Detection System (Bio-Rad) using SYBR Green according to the manufacturer’s protocol. The primer sequences used for qRT-PCR are listed in Table S2. Thermal cycling was performed using the following parameters: 95 °C for 10 min, then 45 cycles of denaturation at 95 °C for 10 s, and extension at 60 °C for 30 s. The threshold cycle number was recorded for each reaction. The threshold cycle value was normalized to that of GAPDH. Each sample was assayed in triplicate and repeated at least 3 times.

### RNA interference

psi-LVRU6P vectors (carrying a puromycin antibiotic resistance gene) containing control and short hairpin–mediated RNA (shRNA) oligonucleotides were purchased from Gene Copoeia (Rockville, MD, USA). The 2 shRNAs sequences are listed in Table S3.

### Lentivirus production and transduction

Lentiviral production and transduction were conducted according to the manufacturer’s instructions (Gene Copoeia). Briefly, lentiviral vectors and LonP1 or control shRNA were packed with the Lenti-Pac HIV Expression Packaging Kit using HEK293T cells and incubated overnight, followed by replacement of the old culture medium with fresh DMEM supplemented with 5% heat-inactivated FBS and penicillin-streptomycin. Titer Boost reagent (1/500 volume) was added to the culture medium and incubated at 37 °C in a humidified incubator with 5% CO_2_. At 48 h after transfection, the supernatants containing lentivirus particles were collected, filtered through 0.45-μm syringe filters (SLHV004SL, Millipore), and used immediately to infect H9c2 cells. To select stably transfected cells, the old medium was replaced by fresh complete medium containing puromycin (2.0 μg/ml) every 3 days until drug-resistant colonies become visible. The knockdown of LonP1 was validated by qRT-PCR and Western blot analysis. Positive clones with stable knockdown of LonP1 were expanded and maintained in medium supplemented with puromycin (2.0 μg/ml).

### Fluorescence detection in cultured cells

For measuring the colocalization of LonP1 with mitochondria, cells were stained with 50 nM MitoTracker Green for 30 min at 37 °C and then treated with 4% paraformaldehyde for 5 min, followed by incubation with mouse anti-LonP1 antibody at 4 °C overnight. Slides were then incubated with Alexa-Fluor-555-conjugated donkey anti-rabbit antibodies (A-31572, Invitrogen) at room temperature for 2 h. Cells were then stained with DAPI for 30 min at room temperature. Finally, fixed or living cells were visualized by confocal microscopy with a Leica TCS SP8 microscope with a 633-numerical-aperture 1.35 oil objective. To determine mitochondrial morphology, 100 cells were randomly selected for quantitative analysis and visually scored into 4 classifications (tubular, short tubular, fragmented, and large spherical).

For ER and mitochondria contact analysis, we used fluorescence to detect the colocalization of ER with mitochondria. Cells were stained with 50 nM MitoTracker Red for 30 min at 37 °C, then treated with 4% paraformaldehyde for 5 min, and incubated with rabbit anti-PDI antibodies at 4 °C overnight. Cells were further incubated with Alexa-Fluor-488-conjugated donkey anti-rabbit antibodies (A-11034, Invitrogen) at room temperature for 2 h, washed, and then stained with DAPI for 30 min at room temperature. Finally, cells were visualized by the GE DeltaVision OMX ultra-high-resolution microscopic imaging system. Images of ER/mitochondria colocalization were acquired from 3D deconvoluted stacks using the Z-stack application. The Pearson correlation coefficient, a well-defined and commonly accepted means for describing the extent of overlap between image pairs, was applied to quantify the degree of colocalization between ER (green) and mitochondria (red). Colocalization (Pearson’s correlation coefficient) was measured by ZEN software using automatic thresholding.

### XF-96 Extracellular Flux Analyzer experiments

The intact cellular OCR of LonP1 stable knockdown and control H9c2 cells was measured using the Seahorse XF-96 Extracellular Flux Analyzer (Agilent Technologies) as described previously [49]. Results were obtained by performing 3 independent experiments with 8 replicates of LonP1 stable knockdown and control H9c2 cells. After the assay was completed, the protein concentration was determined by a BCA protein assay kit to normalize the OCR according to the manufacturer’s instructions.

### Fluorescence-activated cell sorting analysis for ROS and mitochondrial membrane potential

Intracellular ROS levels were measured using the fluorescence probe DCFH-DA according to the manufacturer’s protocol. DCFH-DA diffuses into cells and is deacetylated by esterases to nonfluorescent DCFH, which is trapped inside the cells and rapidly oxidized by ROS (including H_2_O_2_) to form highly fluorescent 2′,7′-dichlorofluorescein (DCF). The fluorescence intensity at 530 nm is proportional to the ROS levels within the cell cytosol. Briefly, Lon stable knockdown H9c2 or vector control cells were trypsinized, washed with DMEM, and incubated with DCFH-DA at a final concentration of 10 μM in DMEM for 30 min at 37 °C, and then the cells were washed 3 times with DMEM. Mitochondrial superoxide levels were detected by MitoSOX staining according to the manufacturer’s protocol (M36005, Thermo Fisher Scientific). Briefly, control and LonP1 stable knockdown H9c2 cells were incubated with 5 μM MitoSOX Red for 10 min at 37 °C and then subjected to flow cytometry analysis. Data are plotted as median fluorescence intensities (MFI). The mitochondrial membrane potential was analyzed by JC-1 staining according to the manufacturer’s protocol. Briefly, LonP1 stable knockdown H9c2 or vector control cells were stained with 2.0 μM JC-1 in complete medium and incubated for 20 min at 37 °C in the dark. To remove excess JC-1, cells were washed once with prewarmed PBS and pelleted by centrifugation. Cell pellets were resuspended by gently flicking the tubes, and 500 μl of PBS was added to each tube. Cell samples were analyzed immediately using a BD Accuri C6 Plus flow cytometer (BD Biosciences). All experiments were carried out at least 3 times independently, with 3 technical replicates in each experiment.

### Statistical analysis

Statistical analyses were performed with Prism software (GraphPad Prism 7.0). Data were analyzed using unpaired 2-tailed Student’s *t* test. *P* value less than 0.05 was considered as significant; ns indicates not significant. Data with statistical significance (^*^P < 0.05, ^**^P < 0.01, and ^***^P < 0.001) are shown in the figures. All values are presented as the means ± SEM, obtained from at least 3 independent experiments.

## Acknowledgments

We thank C. Reichman (Rutgers-New Jersey Medical School) and J. Zhu (Wenzhou Medical University) for comments on the manuscript and members of the B.L., Z.Y., and G.Y. laboratory for technical support and valuable discussions. **Funding:** This study was supported by grants from National Natural Science Foundation of China (91954101, 31771534, 31570772, and 31070710 to B.L. and 81774022 to L.J.), National Basic Research Program of China (973 Program, 2013CB531702 to B.L. and 2013CB531704 to G.Y.), and the Scientific Research Foundation of University of South China (211RJC002 to B.L.). **Author contributions:** B.L., G.Y., and Z.Y. conceived the project and designed the experimental protocols. Y.L., D.H., S.G., and J.X. generated the cardiomyocyte-specific LonP1 knockout mice. Y.L., D.H. L.J., and S.G. performed most experiments. L.J. Y.T., and Y.L. performed electrocardiography and calculations of heart function. B.L., Y.L., D.H., F.S., G.Y., and Z.Y. analyzed the data. D.H., Y.L., Z.S., and C.Y. performed TEM and confocal microscopy assay. X.H., L.J., F.S., L.L., and T.C. assisted with the animal studies. C.K.S., C.Y., Z.S., and J.X. assisted with part of data analysis. B.L., Y.L., F.S., D.H., S.G., and Z.Y. wrote the manuscript. All authors reviewed the manuscript and approved the final version. **Competing interests:** The authors declare that they have no competing interests.

## Data Availability

All data needed to evaluate the conclusions in the paper are present in the paper and/or the Supplementary Materials. Additional data related to this paper may be requested from the corresponding author upon request.

## Supplementary Materials

Supplementary 1 Figs. S1 to S6
Tables S1 to S3
