# Supplementary material for: LonP1 Links Mitochondria–ER Interaction to Regulate Heart Function
Source: Research (Wash D C). 2023 Jun 16;6:0175. doi: 10.34133/research.0175 (PMC10275618; doi:10.34133/research.0175)
Supplement: Supplementary 1 — Figs. S1 to S6 Tables S1 to S3 [file research.0175.f1.docx]

**Supplementary Materials for**

**LonP1 Links Mitochondria-ER Interaction to Regulate Heart Function**

Yujie Li, Dawei Huang, Lianqun Jia, Fugen Shangguan, Shiwei Gong, Linhua Lan, Zhiyin Song, Juan Xu, Chaojun Yan, Tongke Chen, Yin Tan, Yongzhang Liu, Xingxu Huang, Carolyn K. Suzuki, Zhongzhou Yang^*^, Guanlin Yang^*^, and Bin Lu^*^

^*^Address correspondence to: [lubinmito@usc.edu](mailto:lubinmito@usc.edu).cn (B.L.); [yangguanlin945@163.com](mailto:yangguanlin945@163.com) (G.Y.); zhongzhouyang@nju.edu.cn (Z.Y.)

**SUPPLEMENTAL FIGURES**


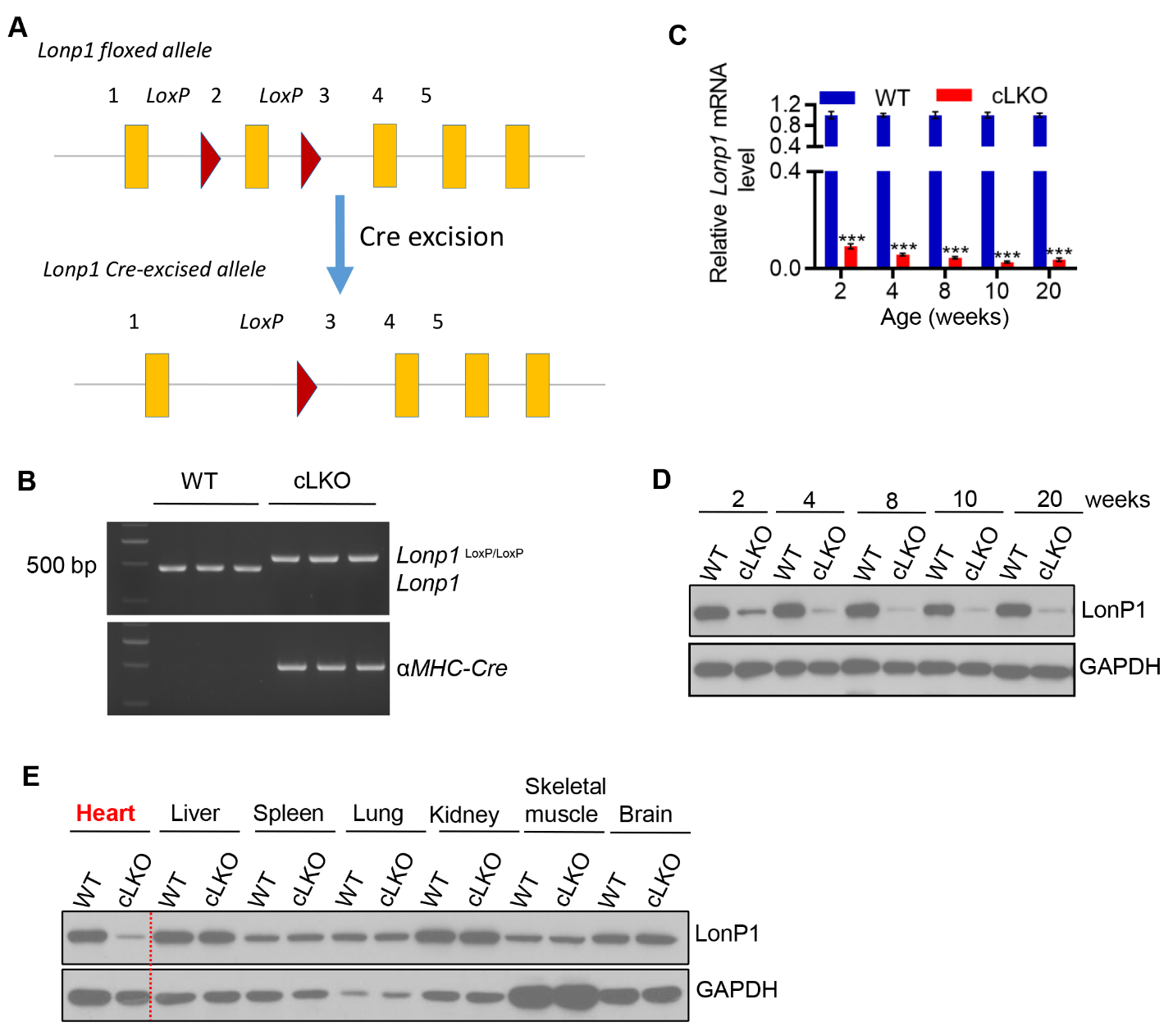


(legend on next page)

**Fig. S1. Generation of Cardiomyocyte-Specific *LonP1* Deletion Mice, Related to Fig. 1.** (**A**) Generation of cardiomyocyte-specific LonP1-deficient mice. *Lonp1^LoxP/LoxP^* mice were crossed with cardiomyocyte-specific-MHC-Cre (*αMHC-Cre*) mice for deletion of LonP1 in the cardiomyocytes. (**B**) PCR products were electrophoresed on a 1% agarose gel. Genotype identification shows *Lonp1^LoxP/LoxP^* alleles located at 573 bp, wild type alleles located at 473 bp, and the α-MHC-cre alleles located in 501 bp. *Lonp1^LoxP/LoxP^* with *α-MHC-cre* refers to cLKO mice. (**C** and **D**) The loss of LonP1 was confirmed at both the transcript (C) and protein (D) levels. Levels of *Lonp1* mRNA and protein were extremely low and almost undetectable in the hearts of LonP1-conditional knockout mice (cLKO) compared with littermate controls (*Lonp1^LoxP/LoxP^*, WT) after two weeks. (**E**) There was no difference in LonP1 expression in the other organs between cLKO and WT mice. In (**C**), data are presented as the means ± SEM (n = 3, ^***^*P* < 0.001).

**
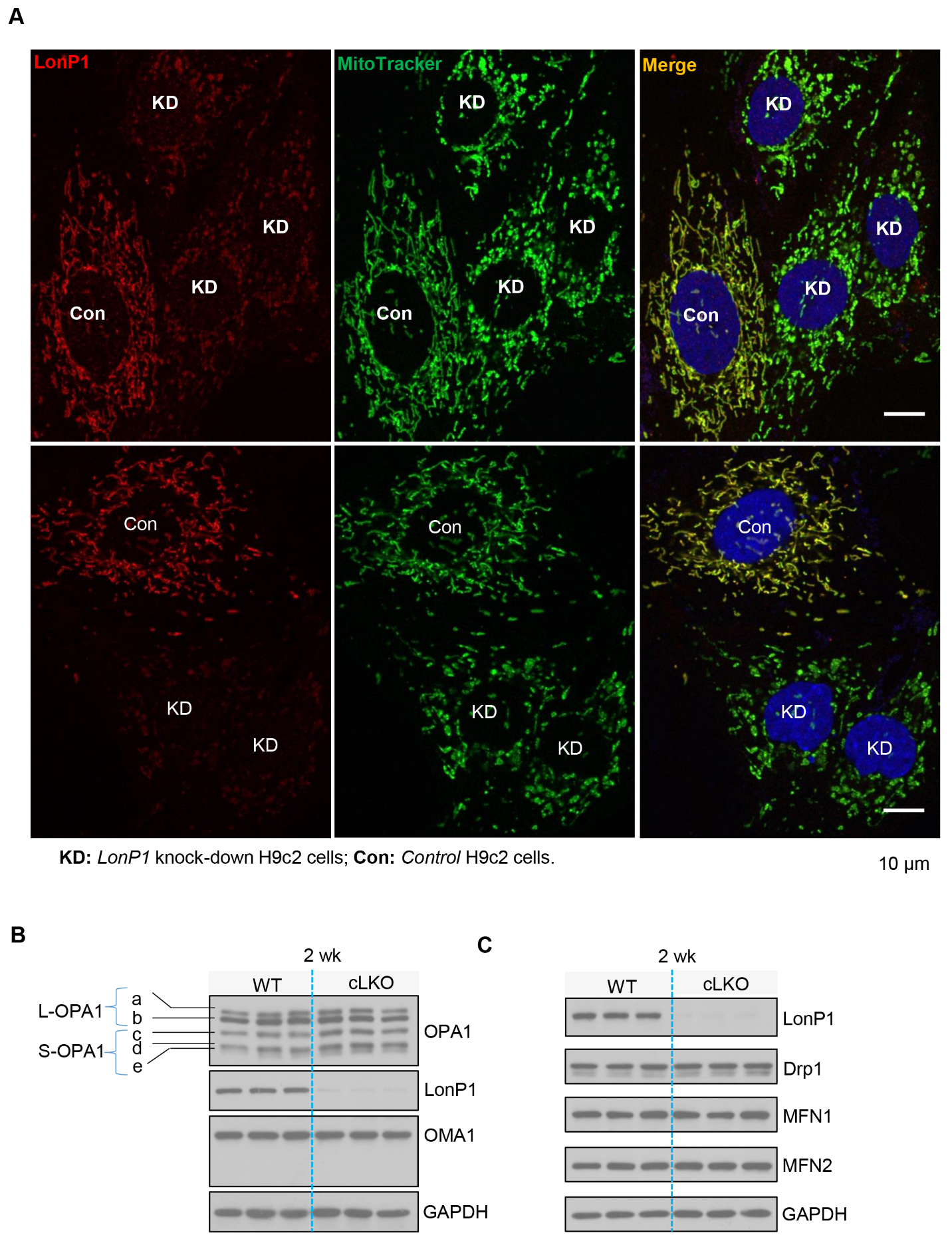
**

(legend on next page)

**Fig. S2. Deletion of LonP1 Leads to Mitochondrial Fragmentation, Related to Fig. 2.** (**A**) Representative confocal microscopy images (scale bars, 10 μm) of mitochondria in *Cont* and sh*LonP1* H9c2 cells. *Cont* or sh*LonP1* H9c2 cells were immunostained with specific LonP1 antibodies and MitoTracker Green. Colocalization of LonP1 and mitochondria was visualized by confocal microscopy. (**B** and **C**) Western blot analysis of mitochondrial fusion- and fission-related proteins OPA1, OMA1, SDHA, Drp1, MFN1, and MFN2, as well as LonP1 protein levels in the heart tissue of WT and cLKO mice at 2 weeks of age. GAPDH was used as a loading control.


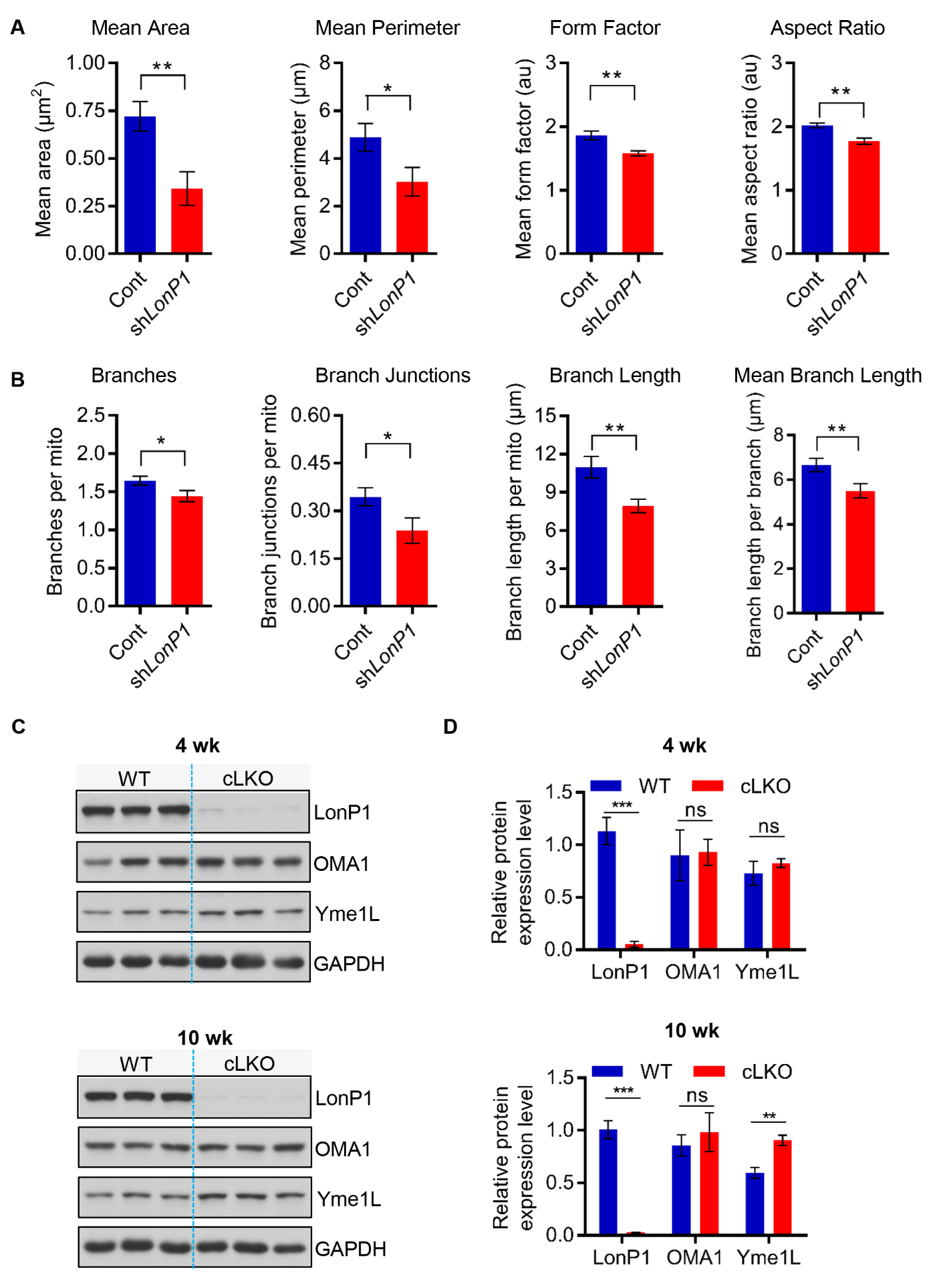
(legend on next page)

**Fig. S3. Deletion of LonP1 Leads to Mitochondrial Fragmentation, Related to Fig. 2.**

(**A**) Quantitative analysis and comparison of mitochondrial morphology in control and LonP1 knockdown H9c2 cells. Data are presented as means ± SEM (n=3, ^*^*P* < 0.05, ^**^*P* < 0.01, statistically significant by Student’s t test). (**B**) Quantitative analysis and comparison of mitochondrial network connectivity in control and LonP1 knockdown H9c2 cells. Data are presented as means ± SEM (n=3, ^*^*P* < 0.05, ^**^*P* < 0.01). (**C** and **D**) Western blot analysis (**C**) and quantification (**D**) of Yme1L, OMA1, and LonP1 protein levels in the heart tissue of 4- and 10-week-old cLKO mice and WT mice. GAPDH was used as a loading control. Data are presented as means ± SEM (n = 3; ns, no significant, ^**^*P* < 0.01, ^***^*P* < 0.001).


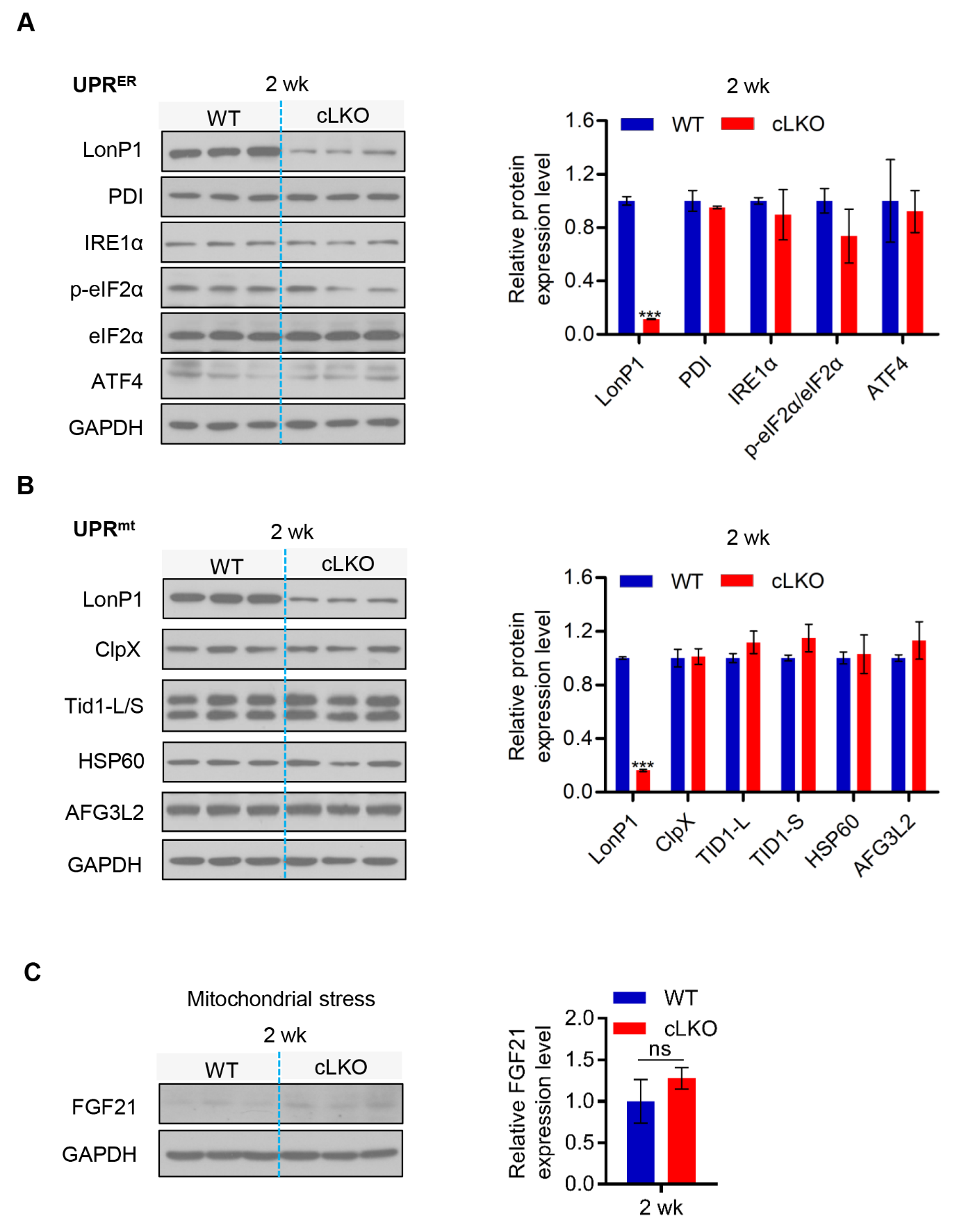


(legend on next page)

**Fig. S4. UPR^ER^- and UPR^mt^-Related Proteins, as well as FGF21 Expression Remained Unchanged in the Hearts of WT and cLKO Mice of 2-Week-Old, Related to Fig. 3.** (**A**) Western blot analysis and quantification of UPR^ER^-related protein levels of PDI, IRE1α, p-eIF2α, ATF4, and LonP1 in the hearts of 2-week-old WT and cLKO mice. GAPDH was used as a loading control. (**B**) Western blot analysis and quantification of UPR^mt^-related protein levels of ClpX, Tid1-L/S, HSP60, FG3L2, and LonP1 in the hearts of 2-week-old WT and cLKO mice. GAPDH was used as a loading control. (**C**) Western blot analysis and quantification of FGF21 level in the hearts of 2-week-old WT and cLKO mice. GAPDH was used as a loading control. In (**A**), (**B**) and (**C**) , data are presented as means ± SEM (n = 3; ns, no significant, ^***^*P* < 0.001).

**;**


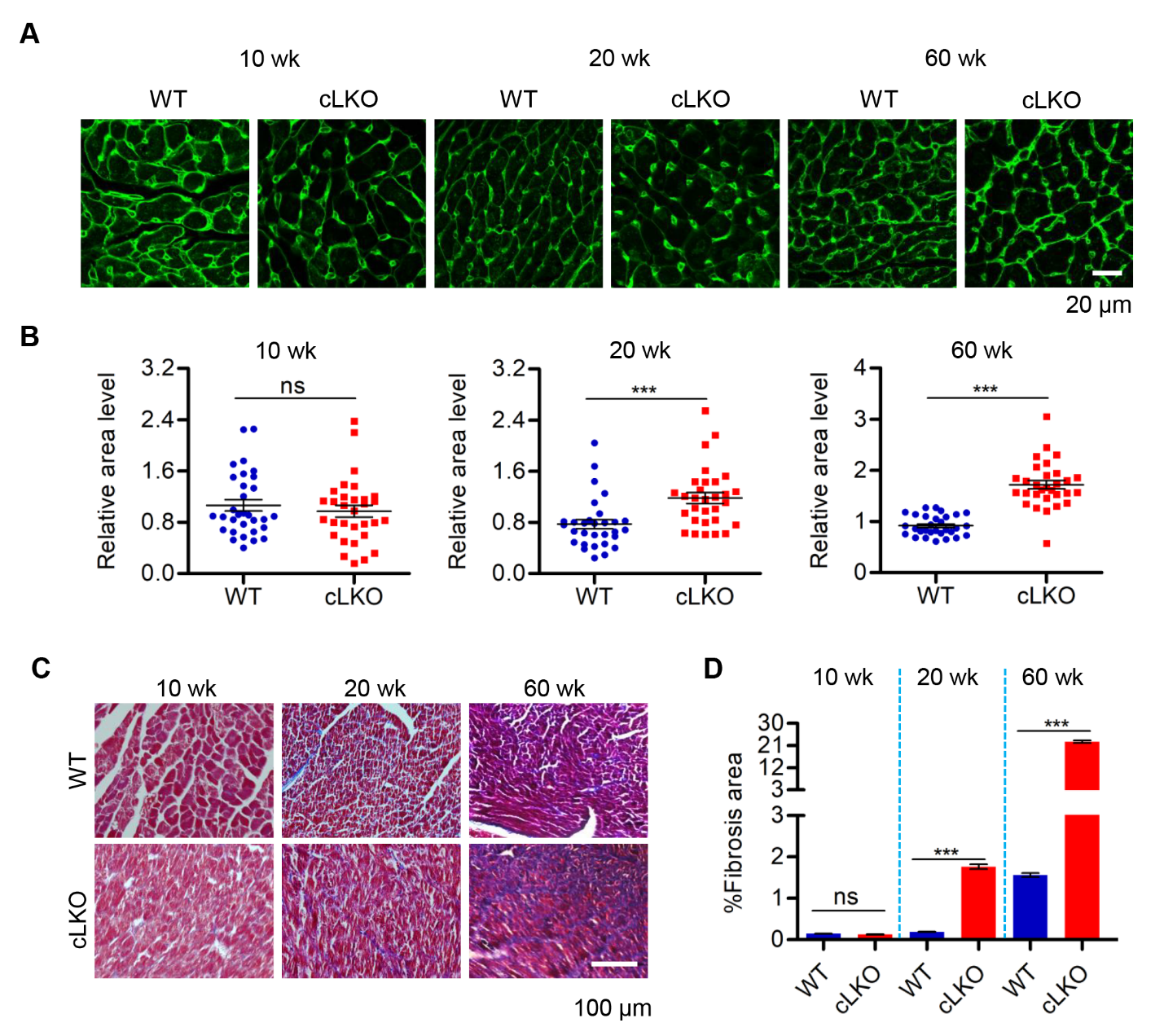


**Fig. S5. Cardiomyocyte-Specific Deletion of LonP1 Causes Pathological Heart Remodeling and Myocardial Fibrosis, Related to Fig. 6.** (**A** and **B**) Wheat germ agglutinin stain to outline the cell surface area in myocardial cell from 10-, 20-, and 60-week old WT and cLKO mice. Scale bars, 20 μm. Data are presented as mean ± SEM (n=30; ns, no significant, ^***^*P* < 0.001, statistically significant by Student’s t test). (**C**) Representative images show Masson’s trichrome staining of the cardiac tissues from 10-, 20- and 60- week-old WT and cLKO mice. Scale bars, 100 μm. (**D**) Quantitative analyses of the heart fibrotic area in 10-, 20-, and 60-week-old WT and cLKO mice. Data are presented as mean ± SEM (n=3; ns, no significant, ^***^*P* < 0.001).


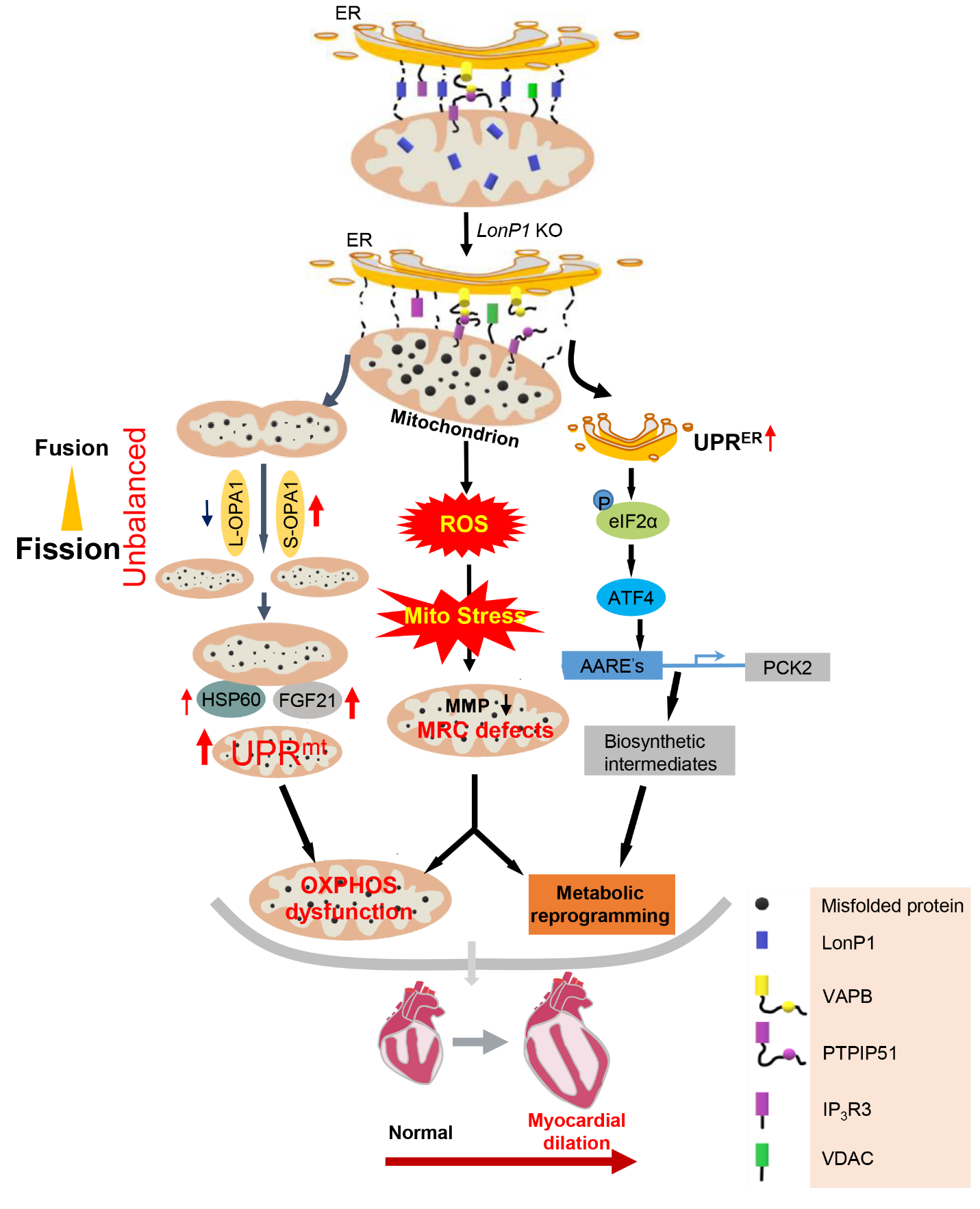


**Fig. S6. Proposed Model of LonP1 in Modulating Heart Functions.**

**SUPPLEMENTAL TABLES**

**Table S1. Genotyping primers of mice. Related to Supplemental Figure 1.**

| **Gene** | **Forward primer** | **Reverse primer** |
| --- | --- | --- |
| ***Lonp1^LoxP/LoxP^*** | 5’-AGGATCACCCTGAGTTCCCA GTT-3’ | 5’-CACCACCTATAGCAGGTGCG AA-3’ |
| ***α-MHC-cre*** | 5’-GCCTGCATTACCGGTCGATG C-3’ | 5’-CAGGGTGTTATAAGCAATCC C-3’ |

**Table S2. Primer Sequence for qRT-PCR. Related to Supplemental Figure 5.**

| **Gene** | | **Forward** | | **Reverse** |
| --- | --- | --- | --- | --- |
| ***Gls*** | 5’-TTCCAGAAGGCACAGACATGG TTG-3’ | | 5’-GCCAGTGTCGCAGCCATCAC-3’ | |
| ***Suclg2*** | 5’-CAGCGAACTTCTTGGACCTTGG AG-3’ | | 5’-TCCGTTGGCAATGATGGCACA G-3’ | |
| ***Pck2*** | 5’-GCGGCTATGGTGGTAACTCCTT G-3’ | | 5’-GCCAGATTGGTCTTGCCACAG G-3’ | |
| ***Phgdh*** | 5’-CAGGTGGTGGAGAAGCAGAAC TTG-3’ | | 5’-GCAGCCTCCAGATCCACATTG TC-3’ | |
| ***Psat1*** | 5’-TCGCTGGTGCTCAGAAGAATG TTG-3’ | | 5’-CTTGATCCATTCCAGGACCAT GCC-3’ | |

**Table S3. shRNA sequences of *LonP1*.**

| ***shRNA*** | | | **Forward** | | **Reverse** | |
| --- | --- | --- | --- | --- | --- | --- |
| *shLonP1* #1 | 5’-GATCCGGCGCTTTATCAAGATCG TGGATCAAGAGCGCGAAATAGTTCTAGCACGTTTTTTTGGAATT-3’ | | 5’-CTAGGCCGCGAAATAGTTC TAGCACGTAGTTCTCGCGCTTTATCAAGATCGTGGAAAAAAACCTTAA-3’ | |  |  |
| *shLonP1* #2 | 5’-GATCCGCGTTCGCTCAGATTCAT GAGATCAAGAGGCAAGCGAGTCTAAGTACTCTTTTTTTGGAATT-3’ | | 5’-CTAGGCGCAAGCGAGTCTA AGTACTCTAGTTCTCCGTTCGCTCAGATTCATGAGAAAAAAACCTTAA-3’ | |  |  |

**SUPPLEMENTAL MATERIALS AND METHODS**

**Construct Method of *Lonp1^LoxP/LoxP^* Mice and Genotyping**

The strain was generated as follows. An L1L2_Bact_P cassette coding FRT-lacZ-loxP-neomycin-FRT-loxP was inserted between exon 1 and exon 2 and another loxP was inserted immediately after exon 2. The linearized construct was transfected into C57BL/6J embryonic stem cells and neomycin-resistant clones were screened by PCR. Clones that had undergone homologous recombination were injected into albino C57BL/6J blastocysts and the resulting chimeric mice were crossed with Gt (ROSA) 26Sortm1 (FLP1) Dym (also known as ROSA26::FLPe knockin) mice to excise the neomycin resistance cassette. The PCR primers for genotyping are listed in Table S1.

**Generation of Heart-Specific *Lonp1*-Knockout Mice and Genotyping**

The cLKO (*Lonp1^LoxP/LoxP^/α-MHC-cre*) mice were generated by crossing *LonP1^LoxP/+^/α-MHC-cre* mice with *Lonp1*^LoxP/+^ mice. Littermate mice were considered as control mice, except the cLKO mice. The mice were maintained on a mixed C57BL/6.SV129 background. No differences between the characteristics of wild-type (WT) *Lonp1*^+/+^ and *Lonp1*^LoxP/LoxP^ mice were observed from birth until more than 1 year old. Genomic DNA was extracted from tail snips using a QIAGEN Gentra Puregene Tissue kit, and PCR was performed using the genomic DNA obtained as the template. The PCR primers for genotyping are listed in Table S1.

**Reagents and Antibodies**

BCA Protein Assay Kit and Pierce ECL Western Blotting Substrate were obtained from Thermo Fisher Scintific. Primary antibodies included anti-MFN1 (13798-1-AP, Proteintech); anti-MFN2 (12186-1-AP, Proteintech); anti-AFG3L2 (14631-1-AP, Proteintech); anti-Yme1L (11510-1-AP, Proteintech); anti-GAPDH (M20028, Abmart); anti-LonP1 (28020S, Cell Signaling Technology); anti-HSP60 (12165, Cell Signaling Technology); anti-PDI (3501P, Cell Signaling Technology); anti-IRE1α (3294P, Cell Signaling Technology); anti-eIF2α (5324S, Cell Signaling Technology); anti-p-eIF2 α (3597S, Cell Signaling Technology); anti-ATF4 (11815s, Cell Signaling Technology); anti-Drp1 (611113, BD-Pharmingen); anti-OPA1 (612607, BD-Pharmingen); anti-Tid-1 L/S (sc-18820, Santa Cruz); anti-OMA1 (sc-515788, Santa Cruz); anti-SDHA (ab14715, Abcam); anti-ClpX (ab168338, Abcam); and anti-FGF21 (ab171941, Abcam). Protease (Complete Mini) and phosphatase (PhosphoSTOP^TM^) inhibitor cocktail tablets were purchased from Roche Applied Science. MitoTracker® Green FM (9074) was purchased from Cell Signaling Technology, and MitoTracker® Red CMXRos (M7512) was obtained from Thermo Fisher Scientific. The trichrome stain kit (ab150686) was purchased from Abcam. Wheat germ agglutinin (W834) was purchased from Thermo Fisher Scientific.

**Wheat Germ Agglutinin (WGA) Staining**

Mouse heart tissues were fixed, embedded, and sectioned as aforementioned. After deparaffinization and rehydration, sections were stained with 20 mg/ml FITC conjugated-wheat germ agglutinin at room temperature for 30 min, washed with PBS three times and sealed with 50% glycerin.

**Masson Trichrome Stain (Connective Tissue Stain)**

Masson trichrome staining was performed using a Trichrome Stain Kit (Abcam, ab150686) according to the manufacturer’s instructions. Briefly, paraffin-embedded heart section was deparaffinized/hydrated, and a series of washes was performed. Then, the slides were placed in preheated Bouin's fluid for 60 min, followed by a 10 min cooling period. Equal parts of Weigert’s (A) and Weigert’s (B) were mixed, and slides were stained with working Weigert’s iron hematoxylin for 5 min, then rinsed slide in running tap water for 2 min. Then, Biebrich Scarlet /Acid Fuchsin solution was applied to the slides for 15 min. The slides were rinsed in distilled water and differentiated in phosphomolybdic/phosphotungstic acid solution for 10-15 min or until collagen was not red. Without rinsing, aniline blue solution was applied to the slides for 5-10 min, and the slides were rinsed in distilled water. Then, acetic acid solution (1%) was applied to the slides for 3-5 min. Finally, the slides were dehydrated very quickly in 2 changes of 95% alcohol, followed by 2 changes of absolute alcohol, then cleared in xylene and mounted in synthetic.

**Western Blot Analysis**

Tissue samples were washed 3 times with ice-cold PBS and homogenized using a homogenizer (Kinematica AG) in 1.5 ml tissue RIPA lysis buffer (50 mM Tris-HCl, pH 7.4, 1.0% Triton X-100, 1% sodium deoxycholate, 0.1% SDS, 150 mM NaCl) supplemented with a protease inhibitor cocktail tablet, and PhosSTOP phosphatase inhibitor cocktail tablets. Tissue homogenates were cleared by centrifugation at 18,000 × g for 25 min at 4°C, and the supernatants were collected in clean microcentrifuge tubes on ice. A similar procedure was used to prepare whole-cell extracts from cells. Briefly, cells were washed with ice-cold PBS and lysed in RIPA lysis buffer supplemented with protease and phosphatase inhibitors on ice for 20 min, followed by centrifugation at 18,000 × g for 30 min at 4°C, and the supernatants were collected. Protein concentrations of the tissue homogenates or whole cell extracts were determined using the Pierce BCA protein assay kit. Tissue or cell extracts equivalent to 20 μg total protein were resolved in 10% SDS-PAGE gels followed by electrophoretic transfer onto a nitrocellulose membrane (Bio-Rad) in Tris-glycine buffer. Blots were blocked at room temperature for 1.5 hours in 5% nonfat milk in Tris-buffered saline (TBS)-Tween (TBST) on a shaker and then incubated with the indicated primary antibodies in 5% nonfat milk TBST overnight at 4°C. The membrane was washed in TBST at least 3 times for 10 min and then incubated with horseradish peroxidase (HRP)-conjugated anti-rabbit or anti-mouse immunoglobulin G at room temperature for 1 hour with gentle shaking. Immunoreactive proteins were detected by ECL reagent according to the manufacturer’s protocol (Thermo Fisher Scientific). The optical density of the Western blot signals was quantified using the National Institutes of Health ImageJ software.

**Protein Extraction and Trypsin Digestion**

Samples were first ground in liquid nitrogen, and then the cell powder was transferred to a 5-ml centrifuge tube and sonicated three times on ice using a high-intensity ultrasonic processor (Scientz) in lysis buffer (8 M urea, 1% Triton-100, 65 mM DTT and 0.1% Protease Inhibitor Cocktail). The remaining debris was removed by centrifugation at 20,000 × g at 4 °C for 10 min. Finally, the protein was precipitated with cold 15% TCA for 2 hours at -20 °C. After centrifugation at 4 °C for 10 min, the supernatant was discarded. The remaining precipitate was washed with cold acetone three times. The protein was redissolved in buffer (8 M urea, 100 mM TEAB, pH 8.0), and the protein concentration was determined with a 2D Quant kit according to the manufacturer’s instructions. For digestion, the protein solution was reduced with 10 mM DTT for 1 hour at 37 °C and alkylated with 20 mM IAA for 45 min at room temperature in darkness. For trypsin digestion, the protein sample was diluted by adding 100 mM TEAB to a urea concentration of less than 2 M. Finally, trypsin was added at a 1:50 trypsin-to-protein mass ratio for the first digestion overnight and at a 1:100 trypsin-to-protein mass ratio for a second 4 hour-digestion. Approximately 100 μg protein from each sample was digested with trypsin for the following experiments.

**iTRAQ Labeling and LC-MS/MS Analysis**

After trypsin digestion, peptides were desalted by a Strata X C18 SPE column (Phenomenex) and vacuum-dried. Peptides were reconstituted in 0.5 M TEAB and processed according to the manufacturer’s protocol for the 8-plex iTRAQ kit. Briefly, one unit of iTRAQ reagent (defined as the amount of reagent required to label 100 μg of protein) was thawed and reconstituted in 24 μl ACN. The peptide mixtures were then incubated for 2 hours at room temperature and then pooled, desalted, and dried by vacuum centrifugation. The sample was then fractionated by high-pH reverse-phase HPLC using an Agilent 300Extend C18 column (5 μm particles, 4.6 mm ID, 250 mm length). Briefly, peptides were first separated with a gradient of 2% to 60% acetonitrile in 10 mM ammonium bicarbonate pH 10 over 80 min into 80 fractions. Then, the peptides were combined into 18 fractions and dried by vacuum centrifugation. Peptides were dissolved in 0.1% FA and directly loaded onto a reversed-phase precolumn (Acclaim PepMap 100, Thermo Fisher Scientific). Peptide separation was performed using a reversed-phase analytical column (Acclaim PepMap RSLC, Thermo Fisher Scientific). The gradient was comprised of an increase from 7% to 20% solvent B (0.1% FA in 98% ACN) over 22 min, then 20% to 35% over 6 min, followed by climbing to 80% over 3 min and holding at 80% for the last 4 min, all at a constant flow rate of 300 nl/min on an EASY-nLC 1000 UPLC system. The resulting peptides were analyzed by a Q Exactive^TM^ Plus hybrid quadrupole-orbitrap mass spectrometer (Thermo Fisher Scientific). The peptides were subjected to an NSI source followed by tandem mass spectrometry (MS/MS) in Q Exactive^TM^ plus (Thermo Fisher Scientific) coupled online to the UPLC. Intact peptides were detected in the orbitrap at a resolution of 70,000. Peptides were selected for MS/MS using the NCE setting of 33, and ion fragments were detected in the orbitrap at a resolution of 17,500. A data-dependent procedure that alternated between one MS scan followed by 20 MS/MS scans was applied for the top 20 precursor ions above a threshold ion count of 2E4 in the MS survey scan with 10.0s dynamic exclusion. The electrospray voltage applied was 2.0 kV. Automatic gain control (AGC) was used to prevent overfilling of the ion trap, and 5E4 ions were accumulated for the generation of MS/MS spectra. For MS scans, the m/z scan range was 350 to 1800. The fixed first mass was set as 100 m/z. The resulting MS/MS data were processed using the Mascot search engine (v.2.3.0). Tandem mass spectra were searched against the UniProt rat rattus database (33,648 sequences). Trypsin/P was specified as the cleavage enzyme, allowing up to 2 missing cleavages. The mass error was set to 10 ppm for precursor ions and 0.02 Da for fragment ions. Carbamidomethyl on Cys, iTRAQ-8plex (N-term) and iTRAQ-8plex (K) were specified as fixed modifications, and oxidation on Met was specified as the variable modification. The FDR was adjusted to < 1%, and the peptide ion score was set at > 20.

**Statistical analysis**

Statistical analyses were performed with Prism software (GraphPad Prism 7.0). Data were analyzed using unpaired two-tailed Student’s t test. P value less than 0.05 was considered as significant; ns, not significant. Data with statistical significance (^*^*P* < 0.05, ^**^*P* < 0.01, ^***^*P* < 0.001) are shown in the figures. All values are presented as the means ± SEM, obtained from at least three independent experiments.
